# Predicting Permissive Mutations That Improve the Fitness of A(H1N1)pdm09 Viruses Bearing the H275Y Neuraminidase Substitution

**DOI:** 10.1128/jvi.00918-22

**Published:** 2022-07-14

**Authors:** Rubaiyea Farrukee, Vithiagaran Gunalan, Sebastian Maurer-Stroh, Patrick C. Reading, Aeron C. Hurt

**Affiliations:** a WHO Collaborating Centre for Reference and Research on Influenza, Peter Doherty Institute for Infection and Immunity, Melbourne, Victoria, Australia; b Department of Microbiology and Immunology, The University of Melbourne, Peter Doherty Institute for Infection and Immunity, Melbourne, Victoria, Australia; c Bioinformatics Institute, Agency for Science, Technology and Research, Singapore, Singapore; d National Public Health Laboratories, National Centre for Infectious Diseases, Ministry of Health, Singapore, Singapore; e Department of Biological Sciences, National University Singapore, Singapore, Singapore; Emory University School of Medicine

**Keywords:** antivirals, influenza, oseltamivir, resistance

## Abstract

Oseltamivir-resistant influenza viruses arise due to amino acid mutations in key residues of the viral neuraminidase (NA). These changes often come at a fitness cost; however, it is known that permissive mutations in the viral NA can overcome this cost. This result was observed in former seasonal A(H1N1) viruses in 2007 which expressed the H275Y substitution (N1 numbering) with no apparent fitness cost and lead to widespread oseltamivir resistance. Therefore, this study aims to predict permissive mutations that may similarly enable fit H275Y variants to arise in currently circulating A(H1N1)pdm09 viruses. The first approach in this study utilized *in silico* analyses to predict potentially permissive mutations. The second approach involved the generation of a virus library which encompassed all possible NA mutations while keeping H275Y fixed. Fit variants were then selected by serially passaging the virus library either through ferrets by transmission or passaging once *in vitro*. The fitness impact of selected substitutions was further evaluated experimentally. The computational approach predicted three candidate permissive NA mutations which, in combination with each other, restored the replicative fitness of an H275Y variant. The second approach identified a stringent bottleneck during transmission between ferrets; however, three further substitutions were identified which may improve transmissibility. A comparison of fit H275Y variants *in vitro* and in experimentally infected animals showed a statistically significant correlation in the variants that were positively selected. Overall, this study provides valuable tools and insights into potential permissive mutations that may facilitate the emergence of a fit H275Y A(H1N1)pdm09 variant.

**IMPORTANCE** Oseltamivir (Tamiflu) is the most widely used antiviral for the treatment of influenza infections. Therefore, resistance to oseltamivir is a public health concern. This study is important as it explores the different evolutionary pathways available to current circulating influenza viruses that may lead to widespread oseltamivir resistance. Specifically, this study develops valuable experimental and computational tools to evaluate the fitness landscape of circulating A(H1N1)pmd09 influenza viruses bearing the H275Y mutation. The H275Y substitution is most commonly reported to confer oseltamivir resistance but also leads to loss of virus replication and transmission fitness, which limits its spread. However, it is known from previous influenza seasons that influenza viruses can evolve to overcome this loss of fitness. Therefore, this study aims to prospectively predict how contemporary A(H1N1)pmd09 influenza viruses may evolve to overcome the fitness cost of bearing the H275Y NA substitution, which could result in widespread oseltamivir resistance.

## INTRODUCTION

Oseltamivir is a neuraminidase inhibitor (NAI) that is prescribed widely for the treatment of influenza and is often stockpiled for pandemic purposes (1–5). This drug was designed to target the conserved active site of the influenza virus neuraminidase (NA) glycoprotein and inhibit its enzymatic function, hence limiting the release of newly synthesized virions from infected host cells (6, 7). However, amino acid substitutions in and around the active site of the NA glycoprotein can reduce virus susceptibility to oseltamivir (8, 9). For example, the H275Y amino acid substitution (N1 numbering) that is commonly reported in the NA of influenza A(H1N1) viruses (1–4, 10, 11) prevents the conformational change of the E276 amino acid which normally creates a hydrophobic pocket necessary for oseltamivir binding, resulting in reduced oseltamivir susceptibility (12–15). Therefore, the emergence of viruses bearing this substitution is of particular concern.

Prior to 2008, the prevalence of the H275Y NA substitution in former seasonal A(H1N1) viruses was generally low (<1%) (11, 16–19). Previous *in vitro* and *in vivo* studies performed with older H1N1 virus strains, such as A/WSN/33 or A/New Caledonia/20/99, showed that variants with the H275Y NA substitution exhibited reduced NA enzyme function, as well as reduced replication and transmission capabilities compared with wild-type viruses (12, 20–24). Given the loss of virus fitness it was assumed that this substitution was unlikely to circulate widely in the community. However, in 2007, an H275Y variant emerged in the A/Brisbane/59/2007(H1N1)-like virus background that was able to outcompete other circulating wild-type strains, and by 2009, almost all circulating viruses carried this substitution (25–29). Fortunately, seasonal A(H1N1) viruses bearing the H275Y substitution were replaced by swine-origin A(H1N1)pdm09 viruses in 2009/2010, and these new viruses retained sensitivity to oseltamivir (30–32). However, the rapid emergence of A/Brisbane/59/2007(H1N1)-like viruses with the H275Y NA substitution highlighted the potential for H275Y variants to be fit and transmissible and demonstrated the need to closely monitor the evolution of the NA glycoprotein of A(H1N1)pdm09 viruses.

To gain insights into the factors facilitating the emergence of a transmissible A(H1N1) H275Y variant, analyses have been performed to compare the effect of the H275Y substitution in the permissive A/Brisbane/59/2007 (H1N1)-like virus background with that of older virus strains (33). These *in vitro* and *in vivo* replication and transmission studies showed that the H275Y NA substitution did not impact the fitness of the A/Brisbane/59/2007(H1N1)-like viruses to the same extent it did to the older virus strains (34–36). Subsequent analyses demonstrated that due to the acquisition of certain substitutions (R222Q, V234M, and D344N), the NA from A/Brisbane/59/2007(H1N1)-like viruses had different enzymatic properties compared with NAs from earlier seasonal A(H1N1) viruses, and these substitutions restored the deficits in NA enzyme function due to H275Y (36–41). Substitutions in the HA (T82K, K141E, and R189K) were also found to play a role in restoring the fitness of A/Brisbane/59/2007(H1N1)-like viruses (42). These studies highlighted that virus evolution can lead to the incorporation of permissive substitutions in viral NA and that they, in turn, have the potential to facilitate the emergence and spread of the H275Y substitution in circulating viruses.

Currently, the prevalence of the H275Y NA substitution in circulating A(H1N1)pdm09 viruses is low (<1%) (1–5, 43). To date, experimental studies assessing viral fitness have shown mixed results, with some reporting comparable fitness between wild-type virus and H275Y variants (44–48), while others report impaired fitness of H275Y variants (49–51). Some clusters of A(H1N1)pdm09 variants with the H275Y substitution have been reported in community settings, notably in Australia in 2011 (52) and in Japan in 2014 (53). A detailed analysis of the viruses from the 2011 Australian cluster demonstrated that the A(H1N1)pdm09 viruses had acquired permissive NA substitutions V241I and N369K, which partially restored the fitness deficit due to H275Y (54, 55). These substitutions (V241I and N369K) are now present in all circulating viruses (54), but given the continued low prevalence of H275Y variants, further permissive substitutions are likely needed for H275Y to become widespread.

Since it is possible for swine-origin A(H1N1)pdm09 viruses to gain permissive mutations for H275Y, our aim was to identify possible substitutions that may emerge in the NA of this virus to facilitate the spread of fit H275Y variants. We have used two different approaches to predict possible permissive NA substitutions in influenza A(H1N1)pdm09 viruses. In our first approach, candidate permissive substitutions were identified using *in silico* calculations to ascertain their impact on protein stability. Our second approach involved the generation of a virus library which was designed to contain every possible single amino acid substitution in the viral NA, while keeping the H275Y fixed. The virus library was then used to infect ferrets via serial transmission to select for variants with high fitness and thereby identify candidates for permissive substitutions. The virus library was also passaged *in vitro* at a low multiplicity of infection (MOI) to select for fit variants. A selection of the candidate NA substitutions identified using either the computational or experimental approach described above were analyzed further to determine their effect on NA cell-surface expression and activity, as well as virus replication. The data obtained from these experiments have allowed us to propose several candidate permissive substitutions for fit H275Y variants and also identify regions of the viral neuraminidase that have a high degree of mutational tolerance and can accommodate such permissive mutations.

## RESULTS

### A computational approach proposed three candidate substitutions which worked synergistically to improve viral fitness.

A computational approach using the FoldX program was employed to identify substitutions that may be permissive for the H275Y substitution in the N1 NA background. This analysis looked at substitutions that co-occurred with H275Y in the NA and were found to occur at least 10 times in influenza databases. There were 25 substitutions identified which were then tested in all possible combinations (up to 4 in each combination) to calculate how they affected the Gibbs free energy required for unfolding the three-dimensional structure of a representative NA. Using them, possible pathways of substitution acquisition were reconstructed *in silico*. Of these substitutions, 15 were shown to improve protein stability *in silico*, and among them, 3 substitutions were chosen as they were observed most frequently in the reconstructed permissive pathways S95N (66 pathways), S299A (99 pathways), and S286G (315 pathways) (see Fig. S1 in the supplemental material).

**FIG 1:**
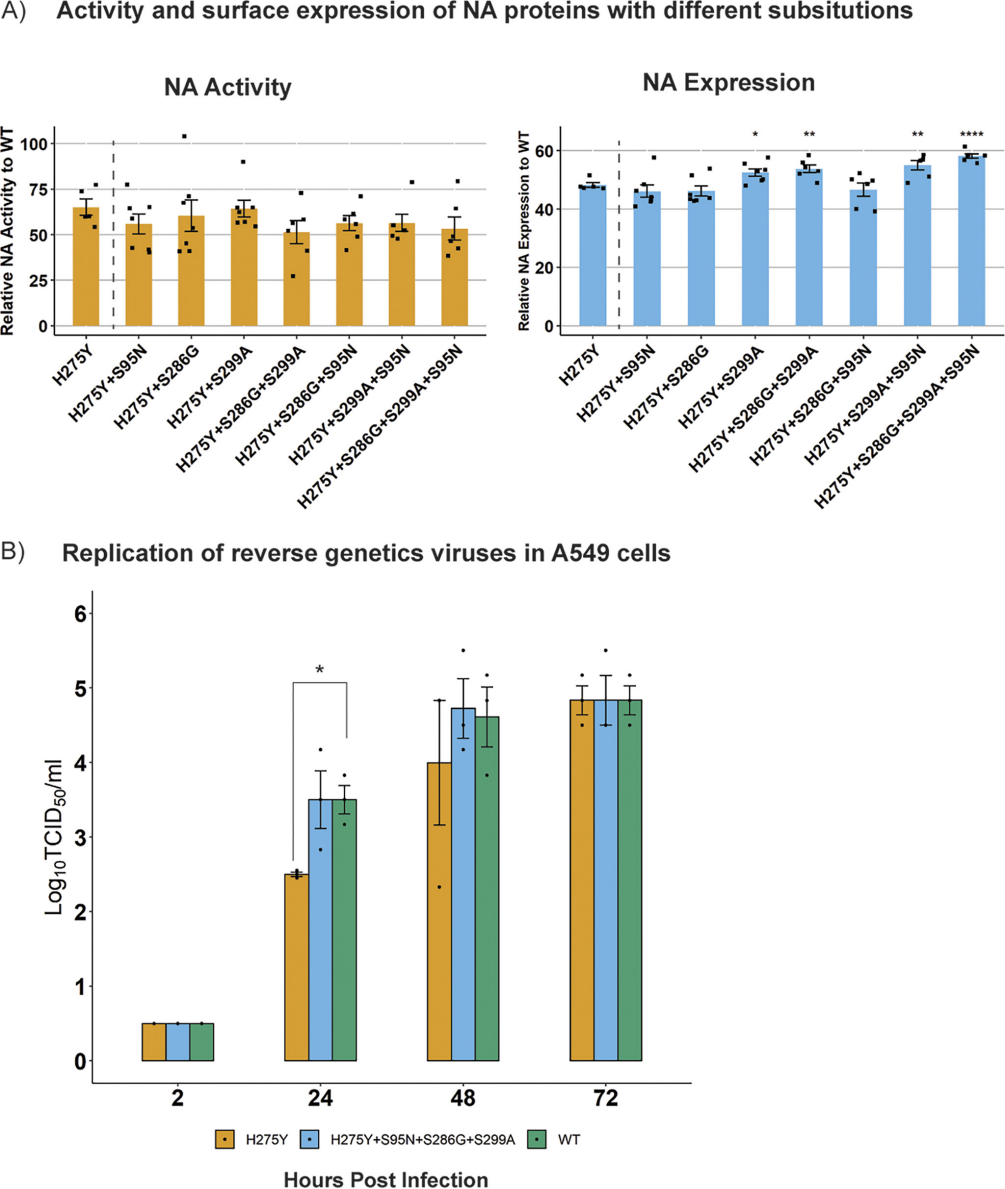
Testing the impact of candidate substitutions derived from computational approaches on NA enzyme function and virus replication. (A) The NA glycoprotein of A/South Australia/16/2017 was mutated such that it expressed the H275Y substitution by itself or in different combinations with candidate permissive substitutions. The proteins were expressed following transfection of 293T cells, and the relative NA activity and expression was calculated as a percentage of the wild-type NA protein. Experiments were performed in duplicate on two separate occasions, and data are expressed as the mean ± SD. The relative NA activity and expression for NA proteins containing candidate substitutions were compared against that of the H275Y-NA using a Student’s unpaired two-tailed *t* test. *, *P* < 0.05; **, *P* < 0.01; WT, wild type. (B) The replication kinetics of reverse genetic viruses, namely, SA16-H275Y, SA16-WT, and SA16-H275Y+S95N+S286G+S299A, was assessed in A549 cells following infection at an MOI of 0.1. The experiment was performed in triplicates and viral titers at each time point measured using a Student’s unpaired two-tailed *t* test. *, *P* < 0.05; **, *P* < 0.01.

The ability of these substitutions to offset the fitness loss due to H275Y was then validated experimentally in a recent A(H1N1)pdm09 viral NA background (A/South Australia/16/2017). First, their impact on NA enzyme function was measured individually and in all possible combinations with each other (Fig. 1). The results showed that the introduction of the H275Y substitution reduced relative NA activity to 65% ± 9% of the wild type, and this activity was not improved substantially by the addition of any of the candidate substitutions (Fig. 1A). The introduction of H275Y also reduced NA expression relative to the wild type (48% ± 2%), but a significant improvement in relative NA expression was observed when the S299A substitution was present, with the greatest increase (10%) observed with the combination of S299A+S286G+S95N (Fig. 1A). However, this increase recovered NA expression only partially relative to the wild type (58% ± 2%), such that expression was still well below 100%.

Since the combination of S299A+S286G+S95N showed the greatest improvement in enzyme expression, the impact of these substitutions on viral growth kinetics was also tested. Replication kinetics in A549 cells demonstrated delayed growth of the SA16-H275Y reverse genetic virus compared with that of the SA16-WT virus, with viral titers reduced at 24 h (2.5 ± 0.0 log_10_ 50% tissue culture infective dose [TCID_50_]/mL versus 3.5 ± 0.2 log_10_ TCID_50_/mL, *P* < 0.05) and 48 h (4.0 ± 0.8 log_10_ TCID_50_/mL versus 4.6 ± 0.4 log_10_ TCID_50_/mL) postinfection (Fig. 1B). Interestingly, the addition of the three substitutions S299A+S286G+S95N recovered this delay in virus growth as observed in Fig. 1B, suggesting a compensatory/permissive role of these substitutions in regaining the loss of viral fitness due to H275Y *in vitro*.

### Deep sequence analysis demonstrates that the SA16-H275Y virus library comprehensively sampled all possible amino acid mutations.

**FIG 2:**
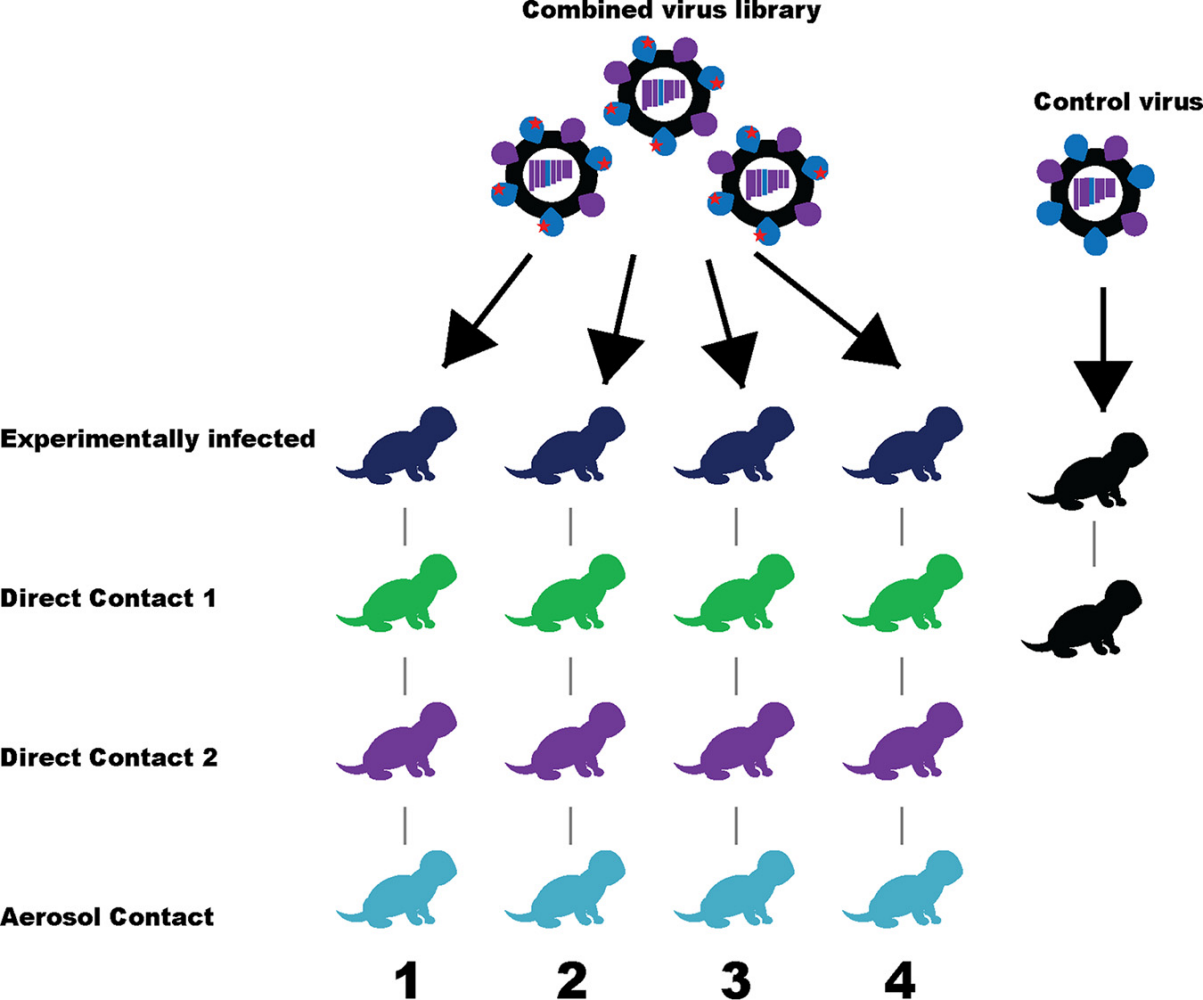
Schematic of the transmission model used to select for fit H275Y variants in the ferret model of influenza infection. Codon-based mutagenesis and reverse genetics were used to generate virus libraries, such that they contained viruses with all possible codon mutations in the A/South Australia/16/2017-NA while H275Y remained fixed. Virus libraries generated on three independent occasions were pooled to increase the likelihood that all codon mutations were represented. The combined library was passaged through ferrets via serial transmission (*n* = 4 independent lines of transmission), and nasal wash samples were collected and analyzed to determine if any variant was selected via passage through ferrets. As a control, the A/South Australia/16/2017-H275Y virus (control) was also generated by reverse genetics and passaged once through ferrets to determine the background mutation frequency.

**FIG 3:**
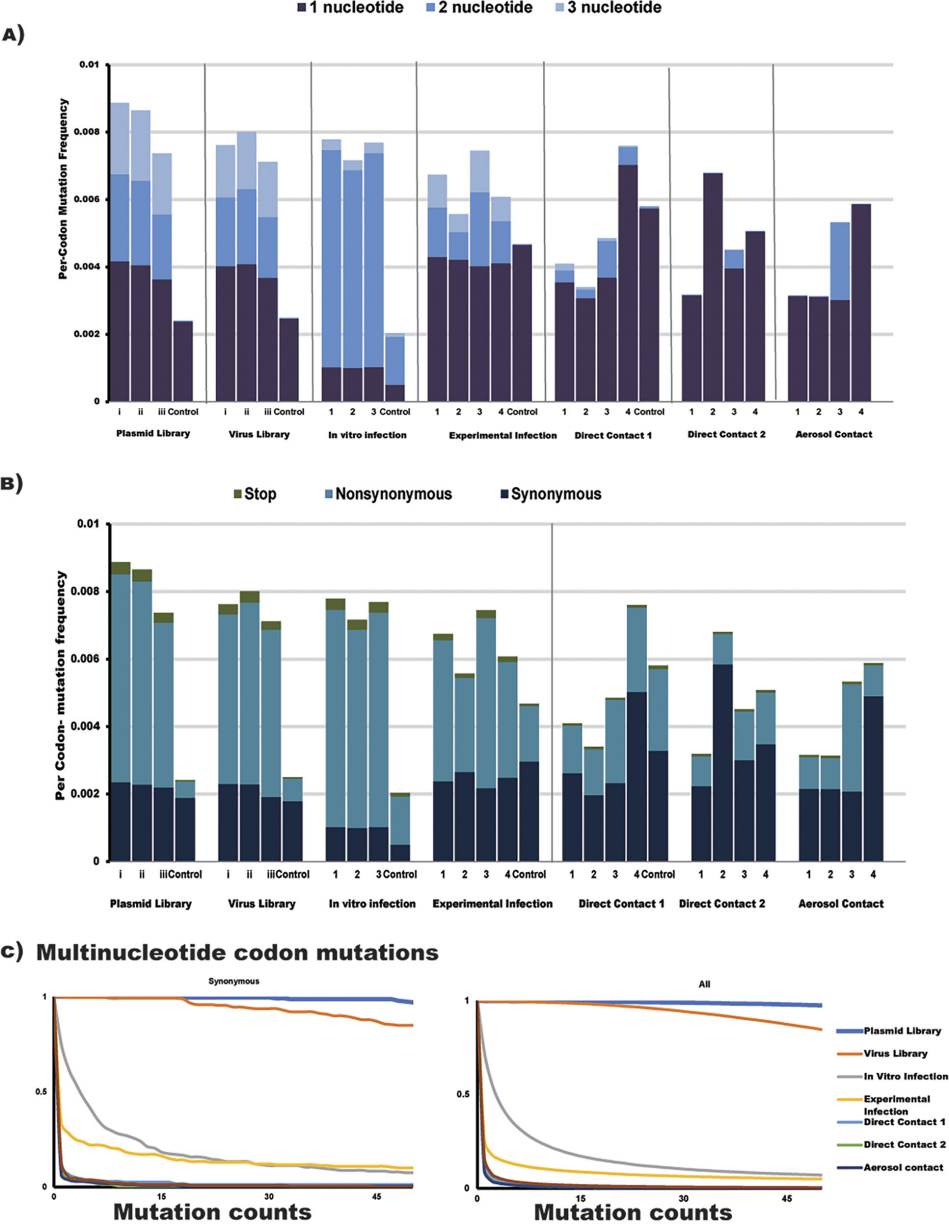
Deep sequencing of plasmid and virus libraries (and relevant controls), as well as ferret nasal wash samples, and viral supernatants after *in vitro* infection was done to determine the per-codon mutation frequency, composition, and fraction of total mutations sampled in the viral NA. (A) Libraries comprised multinucleotide (2 or 3) codon mutations, whereas the controls did not. Compared with the experimental infection of ferrets, most viruses in direct contact 2 and aerosol contact animals contained single-nucleotide codon mutations. (B) Viruses from ferret nasal wash samples generally contained a greater ratio of synonymous changes to nonsynonymous changes, indicating purifying selection. (C) The fraction of multinucleotide mutations that were observed multiple times in the samples, after combining biological replicates, was >90% in the plasmid and virus libraries and was substantially reduced in ferret nasal wash samples and in viral supernatants after *in vitro* infection.

The experimental approach for identifying permissive substitutions involved creating a virus library by reverse genetics (from a NA plasmid library), and then passaging it through ferrets to select for fit variants. This virus library was also made in a recent A(H1N1)pdm09 background (A/South Australia/16/2017). A schematic of the experimental approach is shown in Fig. 2. The virus and plasmid libraries were deep sequenced to test for their completeness in sampling all possible amino acid mutations. An analysis of the reads from these libraries showed that at least 10^7^ overlapping paired-end reads aligned to the NA gene with a codon read depth of at least 10^6^ reads per site, which was adequate to sample all mutations present. The per-codon mutation frequency was substantially higher in the plasmid and virus libraries than that of their respective controls (Fig. 3A and B). The libraries and nasal washes also consisted of one-, two-, and three-nucleotide codon changes, as would be expected from codon mutagenesis. Conversely, the mutations within the controls were almost entirely single-nucleotide codon changes, most likely arising due to sequencing or PCR error (Fig. 3A). The virus library had a slightly lower rate of per-codon mutation than the plasmid library due to the bottlenecking introduced during reverse genetics, and most of the reduction was in the frequencies of nonsynonymous and stop-codon mutations (Fig. 3B).

In order to assess the completeness of the plasmid and virus libraries, the fraction of all multinucleotide codon mutations that were sampled multiple times was also quantified (Fig. 3C). Only multinucleotide mutations were considered, as they are most likely to be introduced due to codon mutagenesis. In previous studies, it was shown that to adequately sample 97% of all possible amino acids in a virus library, only 85% of all possible codon mutations needed to be present at least five times (56). In our study, the combined virus library had more than 99.0% of all multinucleotide mutations sampled at least five times, while the control virus sampled only 3.7% of such mutations at least five times (Fig. 3C).

It should be noted that despite the large diversity of the NA genes in the plasmid and virus libraries, the frequency of each mutated codon in the library was low (0.008% to 0.009%) and the template NA sequence was overrepresented in codon counts.

### Deep sequence analysis of ferret nasal wash samples reveals a stringent bottleneck at each transmission event restricting viral diversity.

**FIG 4:**
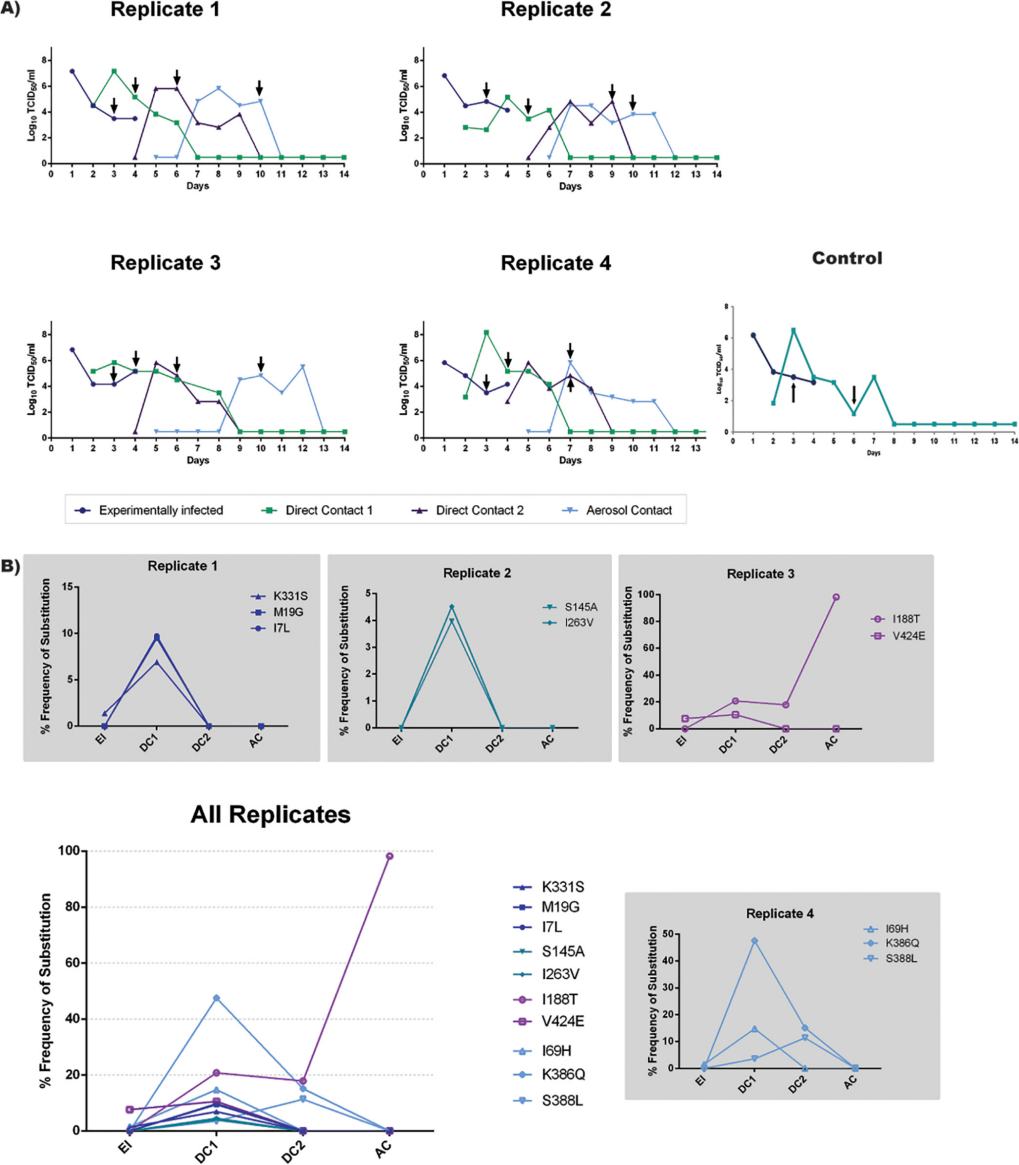
Viral titers and variant frequencies in nasal wash samples from ferrets experimentally infected with the NA-H275Y virus library and from ferrets subsequently infected via transmission. (A) At 24 h postinoculation, experimentally infected animals were cohoused with direct contact 1 ferret. Nasal wash samples from direct contact 1 ferrets were monitored for infection, and on the day that influenza infection was confirmed, they were cohoused with direct contact 2 ferrets. Nasal wash samples from direct contact 2 ferrets were monitored for infection, and on the day that influenza infection was confirmed, they were placed in a cage adjacent to aerosol contacts. All animals were nasal washed daily during the experiment, and infectious virus was detected in nasal wash samples from animals along the transmission chain. For each animal, a single time point (black arrows) was selected for analysis by deep sequencing. (B) NGS data were aligned using Bowtie2, and variants observed at a greater than 1% frequency were called using VarScan, where the average read depth at each site was >10,000 and *P* values for variant calls above 1% were <0.05 for all called positions. A different set of variants were detected in each transmission chain, and most variants were not detected beyond direct contact 1 animals. However, substitutions I188T, K386Q, and S388L were also detected in direct contact 2 animals.

After confirming the completeness of the virus library in sampling all codon mutations, we aimed to investigate whether replication and transmission in ferrets selected for fitter H275Y variants. This experiment was done in replicates of four (Fig. 2). All animals in transmission lines 1, 2, 3, and 4 and control animals were infected successfully as determined by shedding of detectable levels of virus in nasal wash samples (Fig. 4A). In general, recipient or contact animals were found to shed detectable levels of virus within 24 h postexposure to their respective donors. A single nasal wash sample from each ferret was deep sequenced from one time point only (denoted by black arrows in Fig. 4A), based on the rationale described in Text S1 in the supplemental material.

The sequencing results from ferret nasal wash samples were aligned and analyzed in two different ways, as follows: either using a combination of the mapmuts and dms_tools2 pipeline or aligned using the Bowtie2 program and screened for variants using Varscan. At least 10^6^ overlapping paired-end reads could be aligned to the NA genes using mapmuts, and the read depth at each site was greater than 1.5 × 10^5^ reads per site. In contrast, at least 2.6 × 10^7^ reads could be aligned to the NA gene using Bowtie2, and >10^4^ reads per site were used to calculate mutation frequencies and *P* values with Varscan.

There was a trend toward a reduced per-codon mutation frequency along the transmission chain (Fig. 3A and B). Only 13.5%, 2.5%, 1.8%, and 1.9% of all multinucleotide mutations were sampled in viruses from experimentally infected, direct contact 1, direct contact 2, and aerosol contact animals, respectively (Fig. 3C). There was also a greater proportion of single-nucleotide, synonymous mutations and a reduced number of stop codons observed in the aerosol contact animals (Fig. 3A and B). The composition of codon mutations in the animal infected with control virus consisted entirely of single-nucleotide substitutions (Fig. 3A and B).

Nucleotide diversity in the viral populations was also analyzed by calculating π from the Bowtie2 alignment data, which quantified the average number of pairwise differences per nucleotide site. The average π value of viruses from the experimentally infected animals (0.0016 ± 0.0003) was significantly higher than the average π values of viruses from direct contact 2 (π = 0.0006 ± 0.0004) and aerosol contact animals (π = 0.0004 ± 0.0003) (see Table S1 in the supplemental material). Of note, no single nucleotide polymorphisms (SNPs) could be detected by VarScan in animals infected with only the control virus SA16-H275Y, and therefore, no π value is available for these animals.

The ratio between synonymous and nonsynonymous diversity, calculated by πN/πS, was also measured. In general, a πN/πS of <1 indicates a purifying selection that is purging deleterious mutations, a πN/πS of >1 indicates diversifying selection which favors new mutations, and πN/πS of 1 indicates neutrality (57). With one exception, the ratio of πN/πS remained below 1 in viruses from all ferret nasal wash samples (Table S1).

Together, these results demonstrate that there is a significant reduction in viral diversity upon the transmission of influenza virus in ferrets consistent with the presence of narrow bottleneck sizes during transmission. There is also evidence of purifying selection purging deleterious nonsynonymous and stop mutations during virus replication in the ferrets. Of note, the H275Y substitution was not lost during transmission and remained fixed even in viruses from aerosol contact ferrets.

### Bottleneck size estimate reveals a more stringent bottleneck during aerosol transmission than contact transmission.

Given the results described above, it was of interest to learn more about the size of transmission bottlenecks (i.e., the number of transmitting viruses), as it was restricting viral diversity in recipient animals in our study. Utilizing a mathematical model (58), it was calculated that the approximate bottleneck sizes during contact transmission was somewhat varied between each transmission pair, with 24 ± 17 viral particles being transmitted on average (data average across the four replicates in Table 1). However, there was greater variability in estimates of bottleneck sizes during aerosol transmission, where an estimated 146 viral particles were transmitted between one pair (replicate 1), while an average of 7 ± 5 virus particles were transmitted between the three other pairs of ferrets. With a more conservative minimum variant calling cutoff of 3%, the average number of particles being transmitted during contact exposure was 8 ± 6 virus particles and for aerosol transmission was only 2 ± 1 virus particles.

**TABLE 1:** Bottleneck size estimated in donor:recipient pairs using the beta-binomial sampling method

Transmission route	Replicate	Donor^*a*^	Recipient^*b*^	Bottleneck size (1% cutoff)^*c*^	Bottleneck size (3% cutoff)^*d*^
Contact	1	EI	DC1	27 (18, 41)	15 (7, 32)
		DC1	DC2	22 (11, 37)	1 (0, 4)
	2	EI	DC1	60 (38, 90)	19 (5, 107)
		DC1	DC2	4 (2, 7)	2 (1, 2)
	3	EI	DC1	28 (18, 45)	6 (3, 12)
		DC1	DC2	28 (17, 46)	6 (2, 12
	4	EI	DC1	9 (6, 13)	5 (2, 8)
		DC1	DC2	13 (6, 24)	7 (3, 16)
Aerosol	1	DC2	AC	146 (73, 201)	1 (0, 76)
	2	DC2	AC	4 (2, 8)	1 (0, 2)
	3	DC2	AC	5 (2, 8)	2 (1, 4)
	4	DC2	AC	13 (7, 23)	4 (1, 10)

a EI, experimentally infected; DC1, direct contact 1; DC2, direct contact 2.

b AC, aerosol contact.

c Estimated size of bottleneck with lower and upper bounds when a minimum variant calling threshold is set at 1%.

d Estimated size of bottleneck with lower and upper bounds when a minimum variant calling threshold is set at 3%.

### Amino acid substitutions under positive selection pressure in the presence of H275Y in ferrets.

Frequencies of nonsynonymous codon mutations (amino acid substitutions) across the transmission chain were analyzed to see which variants increased in frequency following transmission (Fig. 4B). Variants that increased in frequency following transmission are likely to be under positive selection pressure and hence contain substitutions that may be permissive for H275Y. It should be noted here that each variant in the original library was present at very low levels (0.008%) and was competing with several thousand other variants; therefore, even modest increases in frequency to 4% to 5% can be indicative of a positive selection pressure.

The results reveal that each replicate of the transmission chain was different from the other (Fig. 4B). In replicate 1, M19G (ATG>**GGC**) and I7L (ATA>**C**T**C**) were observed at frequencies of 7% to 9% in direct contact 1 ferrets, despite being below the 1% detection threshold in experimentally infected animals. The substitution K331S (AAG>**TC**G) was observed at 1.4% in experimentally infected animals and rose to a frequency of 6.9% in direct contact 1 animals. Interestingly, the K331S substitution (AAG>**TC**G) was also observed at frequencies of 1.4% to 1.6% in experimentally infected animals from replicates 3 and 4, but these viruses did not transmit to their corresponding recipients (nucleotide changes are highlighted in bold).

In replicate 2, substitutions I263V (ATA>**G**TA) and S145A (TCC>**G**C**G**) were observed at 3% to 4% in direct contact 1 ferrets but were both lost subsequently down the transmission chain. Similarly, in replicate 3, the V424E (GTT>G**AG**) substitution increased from 7.6% in experimentally infected animals to 10% in direct contact 1 animals but was not observed in nasal wash samples later in the transmission chain. The V424E substitution was also observed at a frequency of 5.8% in replicate 4 animals after experimental infection but did not transmit to the corresponding contact animal. The substitution N385D (**A**A**T**>**G**A**C**) in replicate 3 increased from a frequency of 1% in experimentally infected animal to 4.21% in direct contact 1 animals before disappearing altogether. In contrast, I188T (A**TC**>A**CG**) increased from less than 1% in experimentally infected animals to 17% to 20% in direct contact 1 and 2 animals and to 98% in aerosol contact animals.

In replicate 4, substitution K386Q (AAA>**C**AA) increased to 47.5% in direct contact 1 animals but was reduced to 15% in direct contact 2 animals and then was lost altogether in aerosol contact animals. The substitution S388L (TCA>T**T**A) was observed at 3.5% in direct contact 1 animals and 11% in direct contact 2 animals but not in aerosol contact animals. Finally, the I69H (ATC>**CA**C) substitution increased from 1.5% in experimentally infected animals to 14.7% in direct contact 1 animals, before being lost in subsequent animals along the transmission chain. Of note, the I69H substitution was observed in all experimentally infected animals (3% in replicate 1, 1.6% in replicate 2, and 4.5% in replicate 3) but only transmitted to direct contact 1 animals in replicate 4.

As most substitutions were lost following transmission from direct contact 1 animals, it was of interest to sequence nasal washes of direct contact 1 animals across different experimental days to test for the genetic stability of the variants observed in these animals (see Fig. S2 in the supplemental material). The results showed that all the variants observed were stable during the experimental days in direct contact 1 animals and further that V424E increased in frequency from 4% to 19% in replicate 3 direct contact 1 animals. This analysis also revealed two more substitutions in replicate 1 direct contact 1 animal, namely, D451G (GAC>**CTG**) and Y402A (TAT>**GCG**), which were present at 37% and 25%, respectively, and were below the detection limit in the corresponding animal that had been experimentally infected in this line of transmission.

Full genome sequencing revealed that the reversion of the S31N mutation remained stable during transmission events, and while a small number of variants were observed, no sustained changes in the internal genes of the virus were observed (see Table S2 in the supplemental material).

### Evaluation of SA16-H275Y fitness with I188T, K386Q, and S388L.

**FIG 5:**
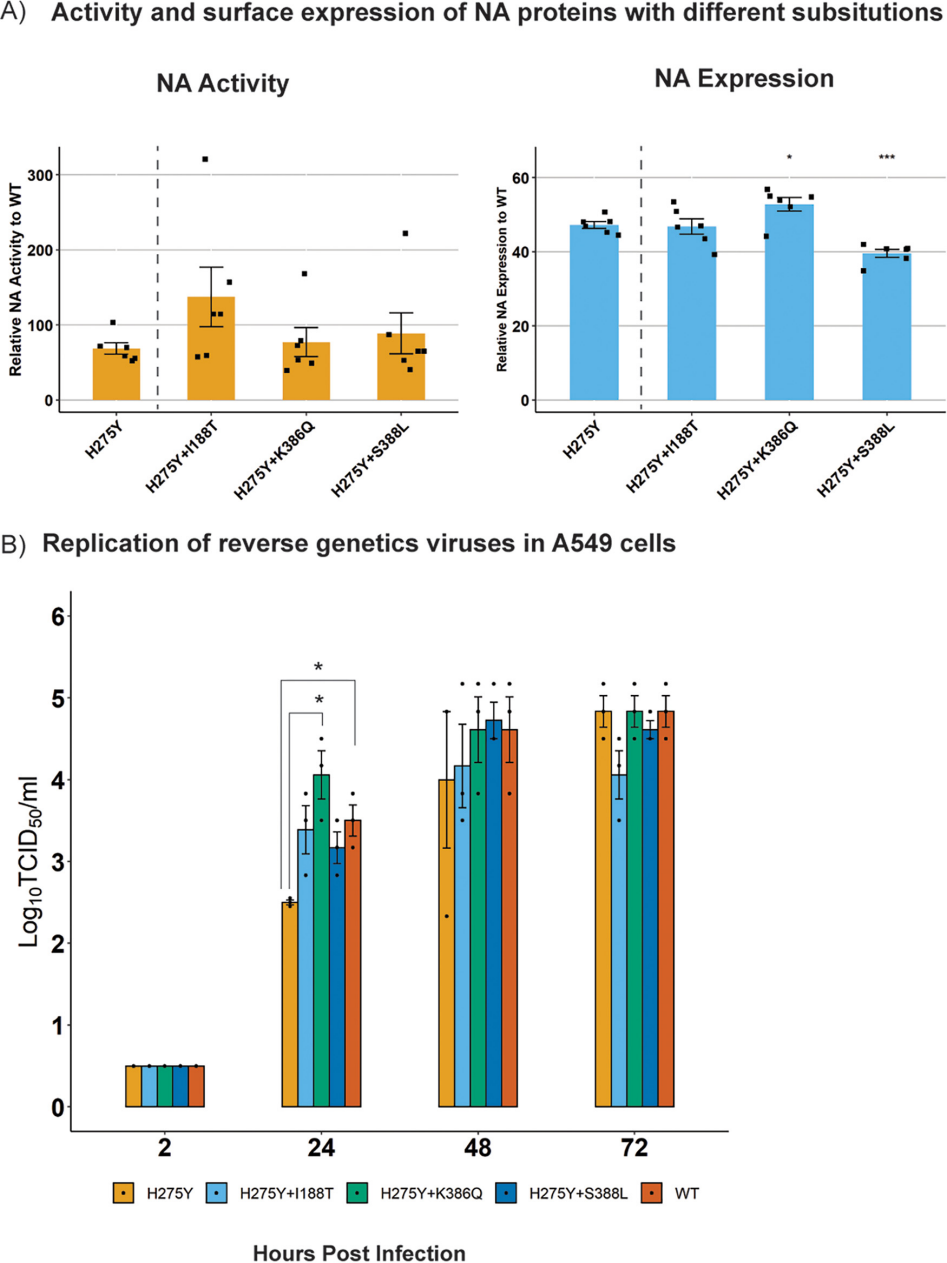
Impact of candidate substitutions identified following passage of virus libraries in ferrets on NA enzyme function and virus replication. (A) The NA glycoprotein of the A/South Australia/16/2017 virus was mutated such that it contained the H275Y substitution by itself or in combination with candidate permissive substitutions. The proteins were expressed following transfection of 293T cells, and the relative NA activity and expression were calculated as a percentage of wild-type (WT) NA protein (lacking any substitution). The assay was performed in duplicate on three independent occasions, and the mean ± SD are shown. The relative NA activity and expression for the NA proteins containing candidate substitutions was compared against that of the H275Y-NA using a Student’s unpaired two-tailed *t* test. *, *P* < 0.05; **, *P* < 0.01. (B) The replication kinetics of reverse genetics viruses, namely, SA16-H275Y, SA16-WT, and SA16-H275Y modified with either I188T, K386Q, or S388L NA substitution, was assessed in A549 cells following infection at an MOI of 0.1. The experiment was performed in triplicates, and viral titers at each time point were measured using a Student’s unpaired two-tailed *t* test. *, *P* < 0.05; **, *P* < 0.01.

As substitutions I188T, K386Q, and S388L were present in direct contact 2 animals, they were analyzed further for their effect on enzyme function in the presence of the H275Y NA substitution. The I188T substitution was of particular interest as it reached a frequency of approximately 98% in the replicate 3 aerosol contact animal. Significant variability was observed in the NA activity assay with a relative NA activity of 77% ± 21% recorded for the H275Y-NA (Fig. 5A). The impact of all candidate substitutions on NA activity and expression was compared with that of the H275Y-NA. Overall, there was a trend toward increased activity in H275Y+I188T-NA and H275Y+S388L NA, with relative NA activities of 116% ± 84% and 87% ± 54%, respectively; however, these increases were not significant. The H275Y+K386Q-NA showed similar levels of relative NA activity (79% ± 38%) to the H275Y-NA.

The relative NA expression of the H275Y+I188T-NA was similar to that of the H275Y-NA (46% ± 4% versus 44% ± 5%). However, relative NA expression was significantly increased in the H275Y+K386Q-NA (50% ± 5%) compared with that of the H275Y-NA. Conversely, relative NA expression was significantly reduced with the H275Y+S388L (40% ± 2%) compared with that of the H275Y-NA (Fig. 5A).

The substitutions I188T, K386Q, and S388L were studied further in an *in vitro* replication kinetics experiment (Fig. 5B). All three substitutions led to moderate improvements in viral titers compared with the SA16-H275Y virus at 24 and 48 h postinfection, with a significant increase in virus titers observed with the SA16-H275Y+K386Q at 24 h postinfection compared with the SA16-H275Y virus (4.0 ± 0.3 log_10_ TCID_50_/mL versus 2.5 ± 0.02 log_10_ TCID_50_/mL).

### Evaluating the mutational tolerance of SA16-H275Y NA.

The stringent bottleneck during transmission between ferrets was a limitation of this study. To address this limitation, the virus library was also passaged *in vitro* at a low MOI to select for functional variants. Similar approaches have been used previously by Doud et al. 2016 and Lee et al. 2018 when analyzing influenza libraries with H1 and H3 mutations (56, 59).

As can be seen from Fig. 3, the per-codon mutation frequency was higher in the viruses analyzed following *in vitro* replication than that of viruses recovered from ferret nasal washes, suggesting a less stringent bottleneck during *in vitro* replication. The number of all multinucleotide mutations sampled was also intermediate compared with that of the virus library and experimentally infected animals (35% sampled at least five times) (Fig. 3C). However, the πN/πS ratio remained below 1 for all samples, suggesting that purifying selection pressures were still present during *in vitro* passaging (Table S1).

**FIG 6:**
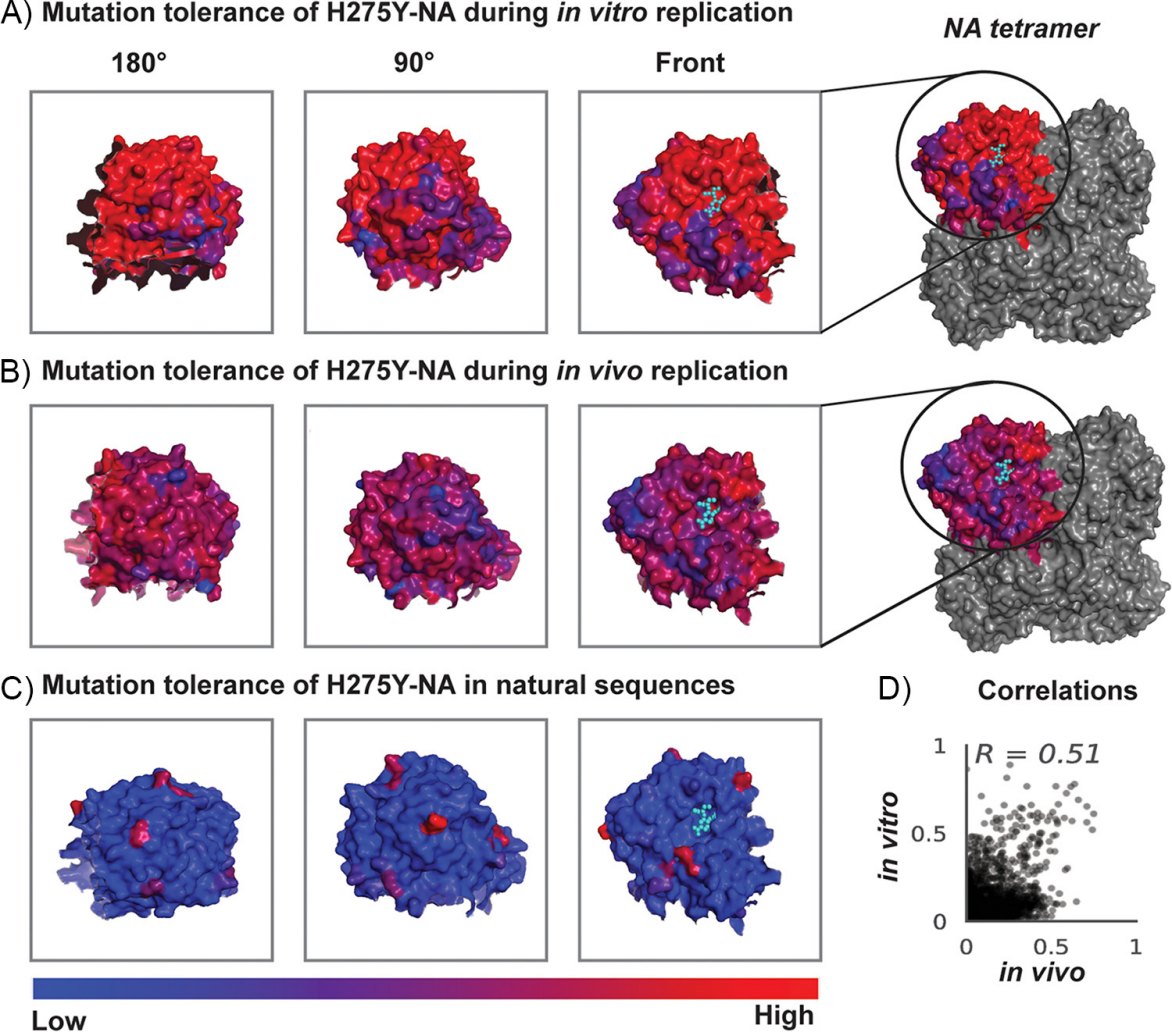
The mutational tolerance of the H275Y-NA glycoprotein in different settings. (A) The amino acid preference (enrichment of amino acid) at each site was calculated from the three replicates of *in vitro* passaging and was used to calculate Shannon entropy (mutational tolerance), which is visualized on the NA monomer using PyMOL (PDB 4B7R). (B) The amino acid preference was calculated from the four replicated of experimentally infected animals, and the Shannon entropy at each site is visualized on the NA monomer. (C) NA sequences from the GISAID acid frequency at each site was used to calculate mutational tolerance at each site. (D) A scatterplot showing correlation between amino acid preferences after *in vitro* replication and amino acid preferences after *in vivo* replication (Pearson’s *r* = 0.51, *P* < 0.01).

It was of interest to use the data from *in vitro* replication to calculate which regions of the H275Y-NA protein had a higher degree of mutational tolerance. Mutational tolerance at each site was measured by calculating the Shannon entropy at each site after measuring the amino acid preferences at each site from the next-generation sequencing (NGS) data. Sites with high variability in amino acid preferences have high Shannon entropy values, which are indicative of high mutational tolerance. Data from *in vitro* replication (Fig. 6A; see Fig. S4 in the supplemental material) and from experimentally infected animals (Fig. 6B, Fig. S4) were used to calculate mutational tolerance during *in vivo* replication. The variability in natural sequences was also of interest, and as such, 639 NA sequences from the Global Initiative on Sharing All Influenza Data (GISAID) database containing tyrosine (Y) in position 275 were aligned using MAFFT, and amino acid frequencies at each site was used to calculate Shannon entropy (Fig. 6C).

**FIG 7:**
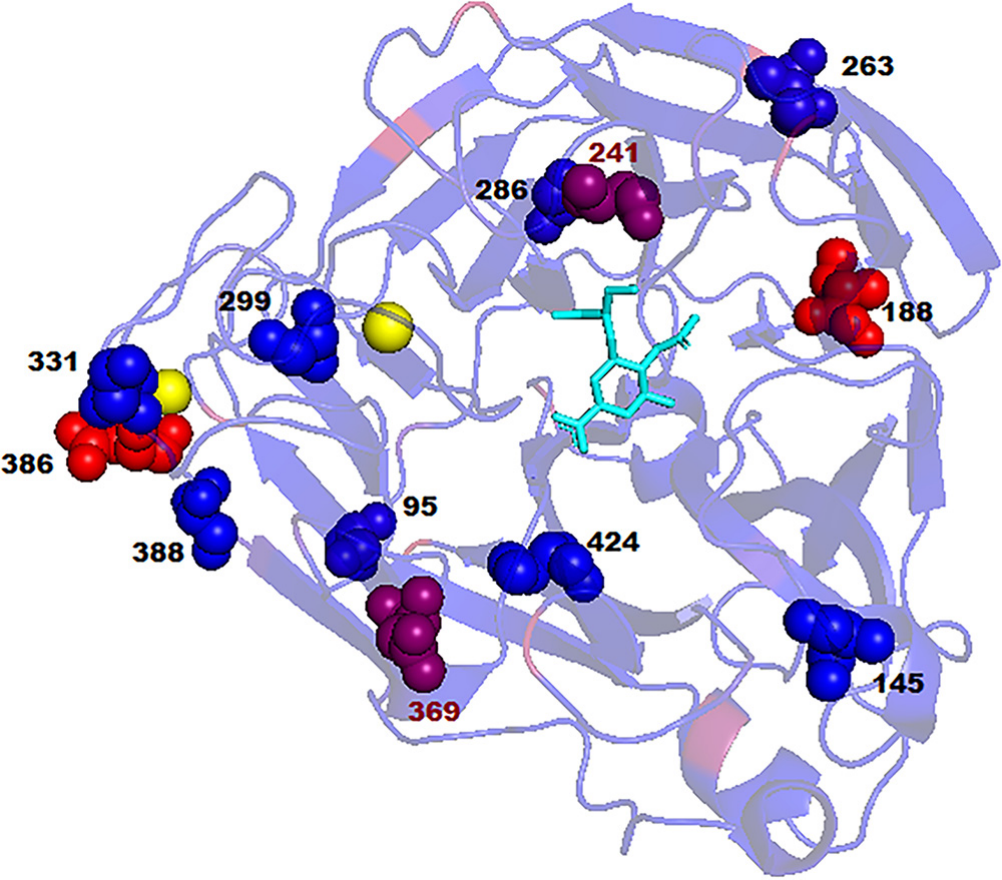
The positions of previously identified permissive substitutions and candidate permissive substitutions identified in our studies have been visualized on a N1 NA monomer from an A(H1N1)pdm09 virus (PDB 4B7R) in complex with oseltamivir. The variability of amino acids at sites of interest was calculated after alignment of all full-length N1 protein sequences from the GISAID database (24,463 sequences). Amino acids are represented on a color scale of blue (low variability) to red (high variability). Oseltamivir is represented as a ligand (cyan), and the two calcium ions in the crystal structure are represented by yellow dots. The positions of previously identified substitutions in N1 NAs, namely, V241I and N369K, are highlighted in red.

The analyses, as visualized on an NA monomer in Fig. 7 and Fig. S4, showed that in *in vitro* settings when there is minimal bottleneck during viral replication, a large number of H275Y variants are able to replicate effectively. This finding suggests that the mutational tolerance of the NA-H275Y is very high, and sites between amino acid 95 to 291 had the highest degree of tolerance compared with other sites. During *in vivo* replication, there are more constraints on viral replication, and as expected, less regions have the relatively high mutational tolerance seen during *in vitro* replication. Calculations showed that sites between amino acid 2 and 12, 43 and 45, 68 and 73, 88 and 91, 121 and 125, 165 and 182, 188 and 191, 199 and 201, 206 and 231, and 259 and 263 had a higher degree of mutational tolerance than other sites (Fig. S4). Encouragingly, there was good correlation in the amino acid preference data derived from *in vitro* and *in vivo* replication (Fig. 7D), suggesting similar sites are variable in both settings. It was also interesting to observe that mutations I69H, T157D, P302H, G320N, and V407F were under strong purifying selection in both settings (blue regions in Fig. S4 logo plots).

In contrast to experimental observations, only 19 sites have shown variability in real life, as seen from the H275Y sequences from the GISAID database (13, 34, 40, 44, 77, 81, 82, 106, 188, 200, 241, 264, 270, 314, 321, 369, 386, 432, and 449). Among these sites, previous analyses have shown that substitutions at positions 241 and 369 were permissive for H275Y.

## DISCUSSION

This study explored two different approaches to predict NA substitutions that may be potentially permissive for the H275Y NA substitution. The first approach utilized predictions of *in silico* protein stability (based on free energy change) to propose candidate substitutions S95N, S286G, and S299A as potentially permissive for H275Y. An analysis of all N1 NA sequences in the GISAID database (34,510 sequences) (Fig. S3) showed that these substitutions occur at a low frequency in natural sequences. The *in vitro* experimental analysis using a 2017 A(H1N1)pdm09 virus background showed that these substitutions in combination with each other (S95N+S286G+S99A) improved NA cell surface expression and offset the reduction in virus titers due to H275Y during *in vitro* replication. In-depth future studies, utilizing *in vivo* models, may shed more light on the impact of these substitution on improving viral fitness. The three mutations are in disparate locations on the NA enzyme (Fig. 7), and therefore, it is unclear which structural interactions may be responsible for them leading to improved NA expression or *in vitro* replication.

The second approach utilized a virus library representing all single NA amino acid substitutions (except H275Y, which was fixed) to select for fit variants during serial transmission in ferrets. A somewhat similar strategy was utilized previously by Wu et al., whereby error-prone PCR was used to generate a virus library with the H275Y substitution, and fit variants were selected after passaging in cell culture (60). However, unlike Wu et al., this study used a more contemporary virus strain (A/South Australia/16/2017 versus A/WSN/33) (60) and performed mutagenesis at a codon level instead of at a single nucleotide level. Moreover, in addition to passaging in cell culture, we have used an appropriate animal model (ferrets) to select for variants with high transmission and replication fitness (60). Finally, none of the variants observed in our study were selected by cell culture passaging in the previous study by Wu et al. (60).

In our study, strong purifying selection was observed in experimentally infected animals and the stringent transmission bottleneck severely restricted viral diversity in the recipient animals. The transmission bottleneck for contact transmission was estimated to allow between 4 and 60 virus particles to transmit between ferrets by contact while 1 to 5 virus particles were transmitted by aerosol transmission (Table 1) (conservative estimate). Our estimates are similar to those proposed from previous studies in ferret and guinea pig models of influenza infection (61, 62) and in a human household transmission study (63).

The substitutions I188T, K386Q, and S388L were of particular interest, as they were detected in nasal wash samples after two transmission events. However, characterizing the effect of these substitutions on NA enzyme function showed that K386Q offset the loss in NA expression due to H275Y by only 5%, and although there was a trend for improved NA activity with I188T and S388L, it was not significant. Reverse genetics viruses with all three substitutions in combination with H275Y showed modest improvements in virus titers following replication in A549 cells compared with viruses with the H275Y substitution alone.

Interestingly, a sequence database analysis of the influenza virus NA revealed that the substitution I188T has increased in frequency from 1.1% in circulating viruses in 2016 to 98% in 2020 (Fig. S3). The strong selection for this substitution in at least one of our replicates using a virus isolated in early (January) 2017 suggests a degree of predictive capability in our experimental analysis. While the high prevalence of I188T in currently circulating viruses suggests that this substitution is unlikely to be fully permissive for H275Y (since H275Y prevalence has not increased since 2016), this substitution is still of interest for further study in combination with other candidate substitutions. The location of this substitution near the oseltamivir/receptor binding site on the NA enzyme (Fig. 7) suggests it may lead to improved viral fitness by stabilizing the structure of the active site. However, further studies will be needed to confirm this idea.

The K386Q substitution is also of particular interest, as previous studies have proposed substitutions at position 386 as candidates for permissive substitutions. For example, a N386S substitution was observed in the NA of a cluster of H275Y A(H1N1)pdm09 variants observed in Newcastle, Australia, in 2011, although it was not present in the majority of the strains circulating worldwide that year (52, 54). The N386K substitution was observed in a cluster of H275Y variants in Sapporo, Hokkaido, Japan, in 2014, and the lysine (K) has since been incorporated into all circulating strains (53). Substitution N386E has been proposed as a permissive mutation in a previous computational analysis (64), and our own computational analysis also identified N386S and N386D as potentially permissive substitutions (Fig. S1). Position 386 therefore appears to be hot spot for amino acid substitutions and could play a key role in viral fitness.

The stochastic nature of transmission placed some limitations on the conclusions we could derive from the ferret transmission study. Therefore, we selected for fit variants after passaging the virus library in cell culture as well. As cell culture replication provided a reduced barrier to the selection of different variants, there was a large diversity of viruses remaining in the resultant virus pools after passaging *in vitro*. It was more useful to utilize these data for a “big picture” analysis by calculating which regions of the NA proteins had a higher mutational tolerance (and were therefore able to accommodate permissive mutations) while H275Y was fixed. It was also interesting to evaluate the mutational tolerance of H75Y-NA after *in vitro* replication compared with tolerance after *in vivo* replication in experimentally infected ferrets. Most interestingly, this analysis showed a good correlation between regions of the NA protein that had a high degree of mutational tolerance in the two settings and identified five substitutions under strong purifying selection (I69H, T157D, P302H, G320N, and V407F).

In summary, utilizing distinct and novel approaches this study has identified several candidate mutations that may be potentially permissive for H275Y. Only a selected number of these mutations were analyzed further, and some mutations showed moderate improvements in viral fitness. While a definite permissive mutation was not identified, this study still provided insights into the regions of the NA proteins where these mutations may arise. It has also provided multiple avenues for further study. For example, the other substitutions that arose in experimentally infected and direct contact animals (Fig. 5) can also be characterized for their impact on viral fitness. Further investigations of the five substitutions under strong purifying pressure in both cell culture and experimentally infected animals, especially I69H, could also be insightful. Moreover, HA sequences of virus recovered from nasal wash samples following passage through ferrets could be analyzed because adaptive substitutions in the HA are also known to restore viral fitness (42). Furthermore, there is also the opportunity to combine the inferences from our experimental approach (where hot spots for mutational tolerances were observed in the NA protein) with our computational approach (through a more exhaustive search for substitutions) to narrow down permissive substitutions. Together, the different approaches utilized here provide insights into the fitness landscape of influenza A(H1N1)pdm09 H275Y variants and present opportunities for further studies, including the development of tools to expedite in-depth studies regarding influenza virus evolution.

## MATERIALS AND METHODS

### Computational approach to predict permissive substitutions: Bioinformatics analysis.

The computational approach included an analysis of N1 protein sequences from human A(H1N1)pdm09 viruses available from Global Initiative on Sharing All Influenza Data website (http://www.gisaid.org) and the influenza virus resource at the National Centre for Biotechnology Information followed by *in silico* protein stability calculations. Briefly, possible substitutions in the potentially permissive pathways were selected through the following criterion: (i) substitutions co-occurred with H275Y in A(H1N1)pdm09 isolates and (ii) minimum frequency of 10 was observed between 2009 and 2012. A selection of 25 substitutions were identified which were then grouped into sets of up to 4 mutations, yielding 12,650 possible combinations representing the serial accumulation of up to 4 mutations in all combinations, in a H275Y background. The RepairPDB and BuildModel commands in the FoldX program (65) were then used to calculate the Gibbs free energy (free energy of unfolding, ΔG, kcal/mol) of a representative three-dimensional wild-type NA protein structure and an NA protein structure containing H275Y, which was derived by homology modeling with Modeller (66) using the NA of A/California/04/2009 (PDB 3NSS) as a template. Potential permissive pathways were constructed representing the serial addition of up to 4 in each group using a custom Perl script. The average change in free energy (ΔΔG) from the wild-type protein was calculated for the H275Y-containing structure and each combination of substitutions in an H275Y background, over 5 BuildModel runs. Permissive pathways were designated a group of mutations for which ΔΔG was observed to be lower than that of the H275Y-containing structure. Fitness threshold based on Gibbs free energy was selected based on previous studies done with H275Y variants from Newcastle, Australia, in 2011 (52).

### Overview of experimental approach for selecting functional variants.

To assess the impact of all possible amino acid substitutions on viral fitness *in vivo*, a virus library was produced that expressed all possible individual codon mutations (2.9 × 10^4^) in the NA while keeping H275Y fixed. The virus library was then passaged through ferrets by serial transmission (*n* = 4 independent lines of transmission) to select for functional variants. Deep sequencing was performed on the virus library to ensure the completeness of the library and on ferret nasal washes on selected days to identify which amino acids were under positive selection pressure in the presence of H275Y. The virus library was also passaged through an MDCK-SIAT-TMPRSS cell line (59) at a low MOI of 0.1 to select for functional variants and sent for deep sequencing.

The virus library was prepared using codon-based mutagenesis and reverse genetics as has been described previously with influenza A HA and nucleoprotein (NP) genes (56, 67, 68). Intermediate steps in creating the library included creating three NA plasmid libraries (i, ii, and iii), followed by three separate virus libraries (i, ii, and iii). The A/South Australia/16/2017 virus isolate (here referred to as the SA16-WT virus) was used as a template for the virus library and was originally submitted to the WHO Collaborating Centre for Reference and Research in Melbourne, Australia, as part of the WHO Global Influenza Surveillance and Response System (GISRS) surveillance program. A detailed description of the library preparation, selection of functional variants in ferrets, passaging in MDCK-SIAT-TMPRSS cells, and the deep sequencing analysis is available in Text S1. A schematic for the selection of functional variants in ferrets is shown in Fig. 2.

To address the biosafety concerns associated with these experiments, the following steps were taken: (i) it was ensured that the A/South Australia/16/2017 backbone is compatible with recent influenza vaccine candidates, such that humoral responses generated to recent vaccines would contain appropriate HA-specific antibodies to recognize the viruses generated; (ii) the S31N substitution in the M2 gene was reverted so that the viruses generated remained sensitive to the antiviral amantadine, and (iii) whole-genome sequencing was performed to confirm that no amino acid substitutions in the polymerase acidic (PA) gene known to confer reduced susceptibility to baloxavir marboxil (E23K/R, I38F/T) were present in any of the viruses generated, thereby implying sensitivity to this antiviral.

### Evaluation of candidate permissive substitutions on viral fitness.

**(i) NA cell surface expression and activity assay.** To gain insights regarding the impact of each candidate substitution identified by computational or experimental approaches described above, we investigated the effect of these substitutions on NA cell-surface expression and NA activity. For these experiments, the H275Y-NA gene from the A/South Australia/16/2017 virus was incorporated into an expression plasmid with a V5 epitope tag and a green fluorescent protein (GFP) signal which was used to measure transfection efficiency. Appropriate substitutions were introduced by site-directed mutagenesis. Measurement of cell-surface NA expression and activity was performed by transfecting 293T cells with the expression plasmid as has been described in our previous studies (37, 54, 64, 69, 70). Of note, this method allowed for cell surface NA activity to be standardized across different samples based on transfection efficiency and cell count.

### (ii) Virus replication in A549 cells.

The most promising candidate substitutions from the previous analyses were incorporated into the SA16-H275Y virus by site-directed mutagenesis and reverse genetics. The replication kinetics of the SA16-H275Y virus, the SA16-WT virus (generated by reverse genetics instead of using the virus isolate to maintain consistency), and the SA16-H275Y viruses with candidate substitutions was then evaluated in a human lung carcinoma epithelial cell line (A549, ATCC CCL 185) following virus infection (MOI, 0.1). The multicycle replication kinetics for each virus was performed in triplicates, and viral titers were determined at 2, 24, 48, and 72 h postinfection.

### Data availability.

All NGS raw data have been submitted to the SRA website and the BioProject accession number is PRJNA561026.
