# Supplementary material for: Predicting Permissive Mutations That Improve the Fitness of A(H1N1)pdm09 Viruses Bearing the H275Y Neuraminidase Substitution
Source: J Virol. 2022 Jul 14;96(15):e00918-22. doi: 10.1128/jvi.00918-22 (PMC9364793; doi:10.1128/jvi.00918-22)
Supplement: Supplemental file 1 — Fig. S1 to S3, Table S1, and Text S1. Download jvi.00918-22-s0002.pdf, PDF file, 1.1 MB [file jvi.00918-22-s0002.pdf]

A

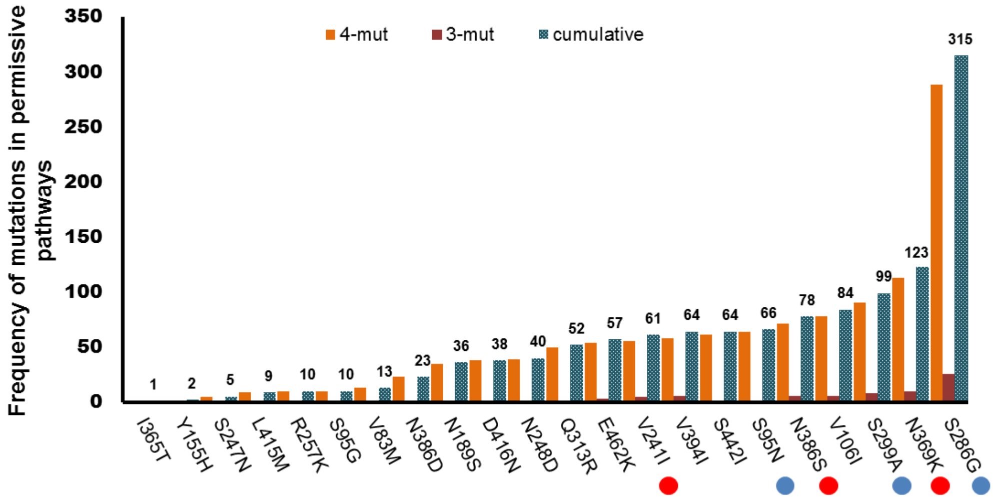

B

Substitution	ΔΔG (kcal/mol)
G382E	3.25
I436V	1.35
S247N	1.35
I374V	0.9
I365T	0.73
Y155H	0.57
V83M	0.43
R257K	0.41
L415M	0.27
S95G	0.26
S286G	-2.05
N369K	-1.04
S299A	-0.92
N386S	-0.8
V106I	-0.75
V394I	-0.57
V241I	-0.52
S95N	-0.45
E462K	-0.45
S442I	-0.3
D416N	-0.18
Q313R	-0.14
N189S	-0.1
N248D	-0.06
N386D	-0.01
H275Y	5.34

Figure S1: Frequency and protein stability of substitution sets in permissive pathways identified by computational analyses of the viral N1 NA. (A) The frequency of the 25 substitutions in the 3 and 4 substitution sets in the permissive pathways. **(B)** The *in silico* effect of each substitution on protein stability was calculated by FoldX, where a negative number represents increased protein stability and a positive number represents reduced stability. For comparison, the effect of H275Y in reducing protein stability was calculated and was shown to reduce in silico protein stability more substantially than any other substitution. The candidate substitutions S286G, S299A and S95N that were selected for experimental analysis are indicated by blue dots (Panel A) or highlighted in blue (Panel B). The permissive substitutions previously evaluated by Butler *et al*.[1] are indicated by red dots (Panel A) or highlighted in red (Panel B). Of note the V106I substitutions was not chosen for further evaluation as it has been replaced with the S200N substitution in currently circulating strains.

Table S1: The nucleotide diversity of the NA gene in viruses from ferret nasal wash samples and after in vitro passaging

Sample from	Replicate No	π	πN/πS
In Vitro passaging	Replicate 1	0.0015	0.5884
	Replicate 2	0.0013	0.5518
	Replicate 3	0.0019	0.5310
	Control	NA	NA
Experimentally infected	Replicate 1	0.0018	0.6261
	Replicate 2	0.0012	0.6046
	Replicate 3	0.0018	0.5296
	Replicate 4	0.0017	0.5299
	Control	NA	NA
Direct Contact 1	Replicate 1	0.0006	0.6372
	Replicate 2	0.0004	0.8021
	Replicate 3	0.0011	0.8182
	Replicate 4	0.0023	0.1975
	Control	NA	NA
Direct Contact 2	Replicate 1	0.0003	1.2227
	Replicate 2	0.0003	0.3404
	Replicate 3	0.0008	0.3786
	Replicate 4	0.0011	0.2736
Aerosol Contact	Replicate 1	0.0003	0.5640
	Replicate 2	0.0002	0.9984
	Replicate 3	0.0002	0.6804
	Replicate4	0.0009	0.0624

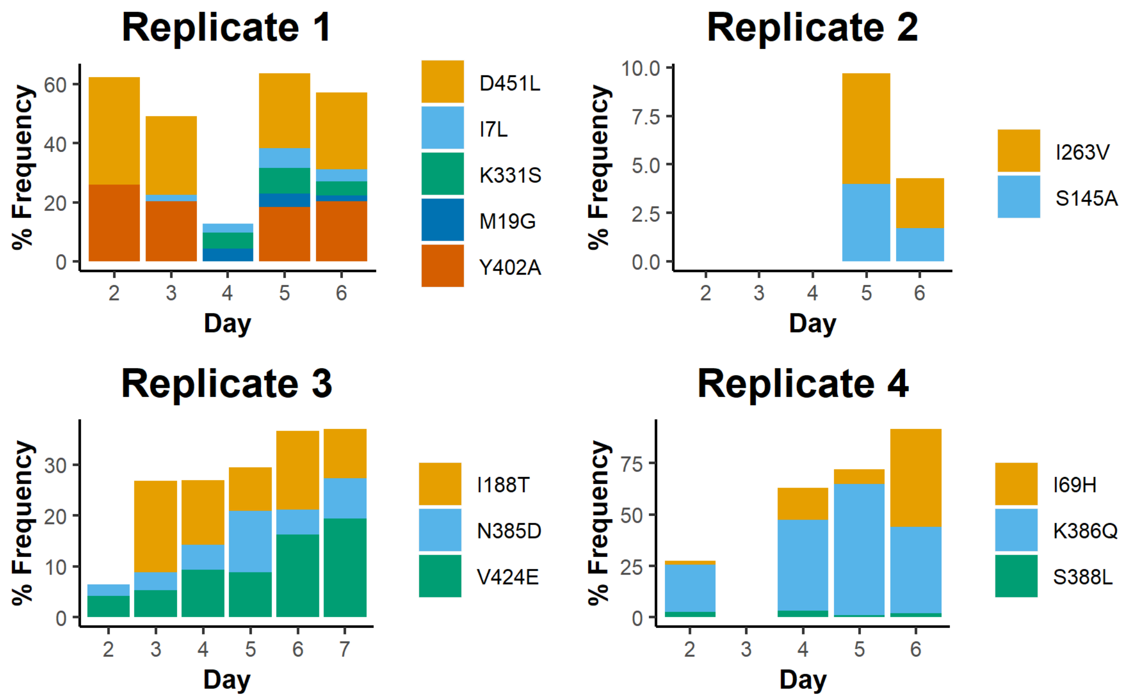

Figure S2: Changes in frequency of variants across experimental days in direct contact 1 animals. Nasal wash samples across the different experimental days were used to extract viral RNA which was then sequenced on the Illumina platform, and aligned using Bowtie2. The frequency of variants was calculated by Varscan. Most direct contact 1 animals stopped shedding infectious virus after day 6/7 and due to poor RNA quality no data is available for day 3 in replicate 4 or days 2, 3, or 4 for replicate 2.

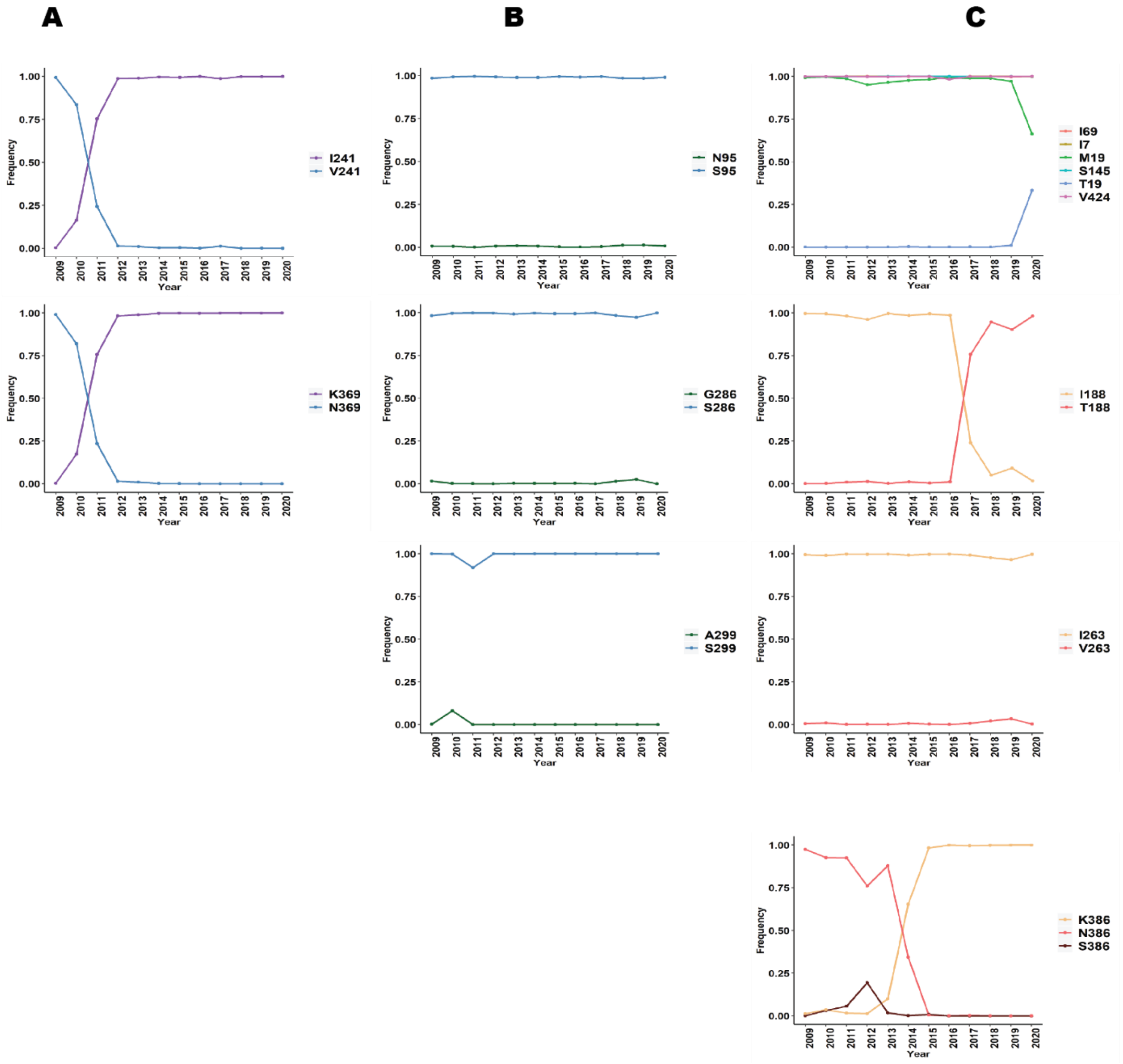

Figure S3: Frequency of previously identified and novel candidate permissive substitutions in circulating N1 NA sequences of A(H1N1)pdm09 viruses. All N1 NA protein sequences submitted to the GISAID sequence database since 2009 were downloaded and cleaned to remove short sequences and sequences with duplicate designations. The 34510 remaining sequences were then aligned using MAFFT and frequency of substitution at each position was calculated using on R. **A)** Frequency of previously identified V241I and N369K permissive substitutions. **B)** Frequency of candidate substitutions proposed by computational analysis and **C)** Frequency of candidate substitutions proposed by experimental analysis.

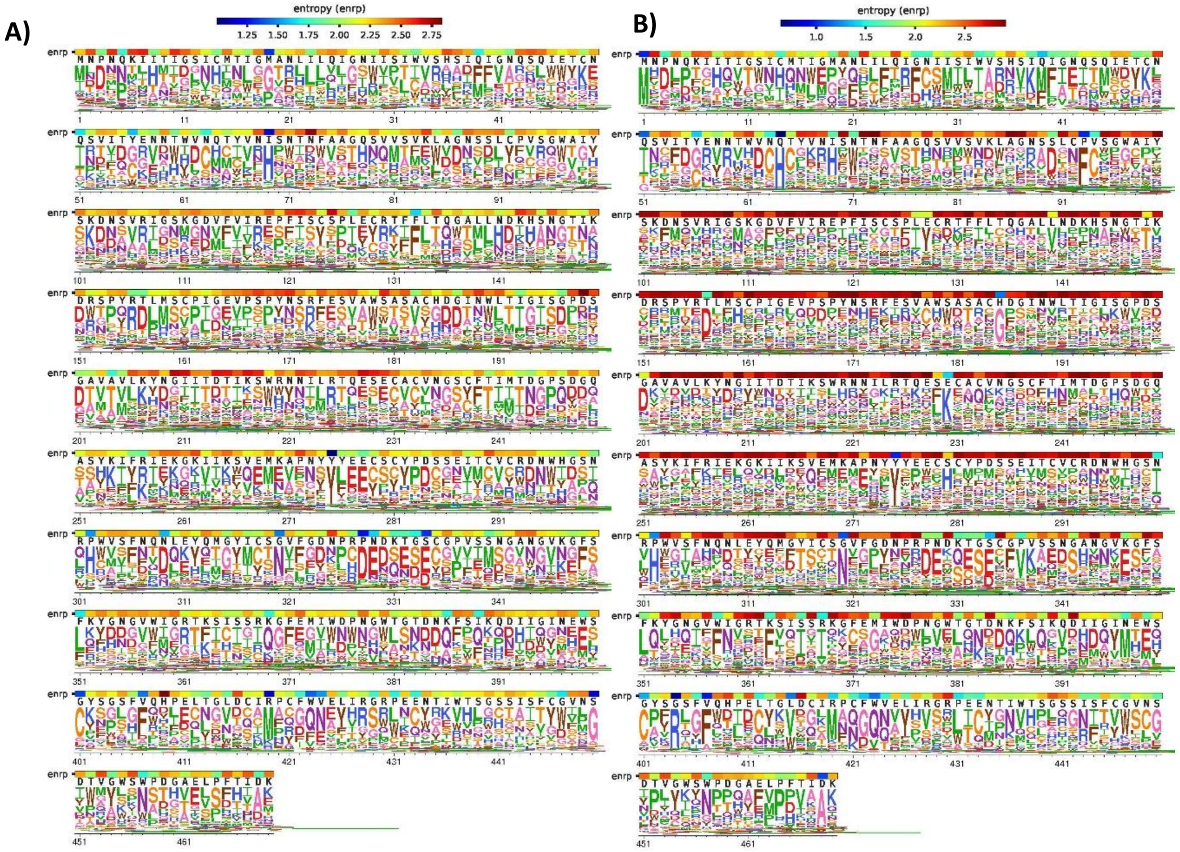

Figure S4: Logoplots showing amino acid preferences after replication of virus libraries under different experimental settings. A) Virus library was used to experimentally infect ferrets, and resultant nasal washes were deep sequenced. By comparing the sequence data before and after selection in ferrets, amino acid preferences at each site could be calculated using the DMStools2 software. Shannon entropy was also calculated from the amino acid preferences, with high values (red) indicating increased mutational tolerance. Logoplots are used to visualized the data. **B)** Virus library was passaged in MDCK-SIAT-TMPRSS2 cells and sequencing data used to calculated amino acid preferences, Shannon entropy and visualized as Logoplots.

1. Butler, J., et al., *Estimating the fitness advantage conferred by permissive neuraminidase mutations in recent oseltamivir-resistant A(H1N1)pdm09 influenza viruses.* PLoS Pathog, 2014. **10**(4).

Supplementary Text S1: Detailed method for preparation of virus library and selection of functional variants in ferrets

Codon Mutagenesis PCR

Codon-based mutagenesis was used to generate a NA plasmid library as has been previously described for influenza A virus HA and NP genes [1-3]. Briefly, mutagenesis primers were designed to introduce randomized NNN nucleotide triplets at each codon site (470 forward and reverse primers were ordered for the 470-residue NA gene), although primers overlapping the H275Y site were not included. The forward and reverse primers were mixed in equimolar ratios to make forward and reverse primer pools. Prior to mutagenesis, template NA from a A(H1N1)pdm09 virus (A/South Australia/16/2017) which expressed the H275Y substitution (H275Y-NA) was amplified from pHW2000-NA plasmid using the Platinum™ *Taq* DNA Polymerase High Fidelity kit (Thermo Fisher Scientific, Australia) and the 5' For-N1 (AACCGGAGTACTGGTCGACCTCCG) and 3' Rev-N1 (GGTATATCTTTCGCTCCGAGTCGGC) primers. PCR products were purified using the QIAquick PCR Purification Kit (Qiagen, Germany). Two low-cycle PCR reactions were carried out on the template NA, one with the forward pooled primers and another with the reverse pooled primers. The PCR reactions contained 15 µL 2x KOD Hot Start Master mix (Merck, USA), 2 µL of either forward or reverse primer pool at final concentration of 4.5 µM, 2 µL of either 5' For-N1 or 3' Rev-N1 primer at 4.5 µM, 4 µL template at 3 ng/µL and 7µL water.

The PCR cycling conditions were as follows:

1. 95 °C for 2:00
2. 95 °C for 0:20
3. 70 °C for 0:01
4. 50 °C for 0:30, cooling to 50 C at 0.5 C/second

5. 70 °C for 0:40
6. Repeat steps 2) to 5) for 6 additional cycles

Following PCR, the forward and reverse fragments were diluted 1:4 in elution buffer and used in a joining reaction. The joining reaction contained 15 µL 2X KOD Hot Start Master Mix, 4 µL of 1:4 dilution of forward fragment, 4 µL of 1:4 reverse fragment, 2 µL 5' N1-For at 4.5 µM, 2µL 3' N1-Rev at 4.5µM and 3µL water. The PCR cycling conditions were the same as described above, but using 20 cycles instead of 7. The joined products were purified, diluted to 3 ng/µL and used as a template for a second round of mutagenesis and joining PCR reactions. The joined products from the second round were then used for ligation.

Gibson assembly for preparing plasmid library

The NA PCR library was utilised to generate an NA plasmid library via Gibson assembly and high efficiency transformation. The pHW2000 plasmid was linearized by BsmBI enzyme digestion and dephosphorylated with Thermosensitive Alkaline Phosphatase (Promega, USA). The assembly for the joined products were set up in duplicates, where 100 ng of purified vector was incubated with 3-fold excess of joined PCR product and 10 µL of 2x Gibson Assembly Master Mix (New England Biolabs, USA), in a total reaction volume of 20 µL. The reactions were incubated at 50°C for 60 minutes, pooled together in a total volume of 40 µL and purified with AMPure XP Beads (bead to sample ratio = 1.8) (Beckman-Coulter, USA).

The purified assembled products were transformed into high efficiency electro-competent MegaX DH10B™ T1R Electrocomp™ cells (Thermo Fisher Scientific, Australia). Briefly, 2.5 µL of assembled products were mixed with 20 µL of bacterial cells and transferred to a 0.1 cm chilled cuvette and electroporation was performed at 2 kV. The bacterial cells were immediately resuspended in 250 µL SOC media and incubated for 1 hour at 37°C, shaking at 220 rpm, and then 200 µL was plated onto agar plates. A 1/2000 dilution of the transformed bacterial cells

were also plated out for colony counting. At least five independent transformations were carried out for each preparation of the assembled products. The following day there were between 200,000 to 400,000 colonies on each agar plate, as determined by colony counting from the diluted agar plates. After pooling colonies from the undiluted agar plates (2,000,000 x 5 = 10^6 colonies total), they were cultured for 4 hours at 37°C and then maxi-preps were performed using the EndoFree® Plasmid Maxi kit (Qiagen, Germany).

To gain insight regarding the distribution of codon mutations in the plasmid libraries, a total of 54 clones were picked from the diluted agar plates (with equivalent numbers of clones taken from the three replicates), and Sanger sequencing was performed. The distribution of codon mutations in the Sanger-sequenced clones were further analysed using a custom python script (https://github.com/jbloomlab/SangerMutantLibraryAnalysis).The three plasmid libraries (i, ii, and ii) were deep sequenced to determine if all possible substitutions had been comprehensively sampled.

Reverse genetics for generation of virus library

Each NA plasmid library was used for reverse genetics to generate a virus library (Figure 1C) [2, 4, 5]. Co-cultures of 293T and MDCK-SIAT1-TMPRRS2 [6] cells in 6-well plates were transfected with pHW2000 containing seven genes from A(H1N1)pdm09 virus A/South Australia/16/2017, and with different NA plasmid preparations containing H275Y-NA from the same virus. Overall, four viral rescues were performed: three with NA plasmid library replicates and one control rescue with the non-library H275Y-NA plasmid. In order to control for loss of viral diversity due to bottlenecks introduced during reverse genetics, each rescue was done in replicates of six and the supernatants from the replicates were then pooled together to create each virus library.

Prior to transfection, $4x10^5$ 293T cell and $0.5x10^5$ MDCK-SIAT-TMPRSS2 cells were seeded in 6-well plates and grown for 24 hours in GIBCO Opti-Mem® + GlutaMax (Life technologies, US) supplemented with 5% (v/v) foetal bovine serum (JRH Biosciences, US), 2 µM L-glutamine (SAFC Biosciences, US), 200 U/mL penicillin (Sigma-Aldrich, US), 200 µg/mL streptomycin (Sigma-Aldrich, US), 0.02M HEPES(SAFC Biosciences) and 2 mg/L Amphotericin B Fungizone (Sigma-Aldrich, US). Viral rescues were performed with equal amounts of the NA plasmid library and the seven internal genes of the A/South Australia/16/2017 virus (HA, NP, NS, M, PA, PB1, PB2) in the pHW2000 plasmid. Growth media was removed from each well and transfected with a total of 1µg of plasmid pre-mixed with 1 ml GIBCO Opti-Mem® + GlutaMax and 18 µL FuGENE® HD transfection reagent (Promega, US). Co-cultures were incubated for 6 hours at 37°C, 5% CO_2 with the transfection mixture, which was then replaced with 1 ml of GIBCO Opti-Mem® + GlutaMax (Life technologies, US) supplemented with 200 U/mL penicillin (Sigma-Aldrich, US), 200 µg/mL streptomycin (Sigma-Aldrich, US) and 0.02 M HEPES(SAFC Biosciences). After another 24 hour incubation, 1ml of GIBCO Opti-Mem® + GlutaMax (Life technologies, US) supplemented with 4 µg/mL TPCK-trypsin (SAFC Biosciences, US), 200 U/mL penicillin (Sigma-Aldrich, US), 200 µg/mL streptomycin (Sigma-Aldrich, US) and 0.02M HEPES(SAFC Biosciences) was added to each well. After a further 48 hour incubation, the supernatants in each well were tested for presence of virus using a standard hemagglutination (HA) test [7]. If HA titre was detected, the supernatant from the replicates in each rescue were pooled, centrifuged at 2000 rpm to remove cell debris and stored at -80 °C. Titres of infectious virus in the virus library preparation were determined using a $TCID_{50}$ assay [8].

The A/South Australia/16/2017 virus isolate (herein referred to as the SA16-WT virus), was submitted to the WHO Collaborating Centre for Reference and Research in Melbourne, Australia as part of the WHO GISRS surveillance programme.

Serial Transmission of virus library in ferrets

Ethics statement

Experiments using ferrets were conducted with approval from the Melbourne University Animal Ethics Committee (project license number 1714278.) in strict accordance with the Australian Government, National Health and Medical Research Council Australian code of practice for the care and use of animals for scientific purposes (8th edition). Animal studies were conducted at the Bio Resources Facility located at the Peter Doherty Institute for Infection and Immunity, Melbourne.

Ferrets

Outbred adult male and female ferrets older than 6-months and weighing 608–1769 g were used. Prior to inclusion in experiments, serum samples were collected and tested by hemagglutination inhibition assay [9] against reference strains of influenza A and B viruses to ensure seronegativity against currently circulating influenza subtypes and lineages. Ferrets were housed individually in high efficiency particulate air filtered cages with *ab libitum* access to food, water and enrichment equipment throughout the experimental period. Ferrets were randomly allocated to experimental groups.

The three virus libraries (i, ii and iii) generated by reverse genetics were pooled into a single virus library to increase the likelihood that all possible substitutions were comprehensively sampled in the final virus library. This final library was subsequently passaged through ferrets in 4 independent lines of transmission to select for variant viruses with the greatest fitness.

Four ferrets were experimentally inoculated with 500 µL containing $10^{4.7}$ $TCID_{50}$ of pooled virus library (day 0), as previously described [10]. One ferret was experimentally inoculated with the SA16-H275Y virus as a control. Each, experimentally infected ferret was then co-housed with a

naïve contact recipient (direct contact 1) 24 hours post-inoculation. Nasal washes were performed daily on direct contact 1 ferrets and nasal wash samples were analysed for infection by qPCR [11]. On the first day that nasal wash samples from direct contact 1 ferrets were qPCR positive for influenza virus, the animal was removed from the cage, and co-housed with a second naïve recipient (direct contact 2). Similarly, nasal wash samples from direct contact 2 were monitored for influenza virus. On the first day that nasal wash samples from direct contact 2 ferrets were qPCR positive for influenza virus, these animals were placed in aerosol cages, adjacent to a third set of naïve recipients (aerosol contacts). Due to limited animal numbers, the SA16-H275Y virus was only passaged once through ferrets (Experimentally infected animals to Direct Contact 1).

In the experiments described, ferrets were nasal washed every day and weight and body temperatures were collected as previously described [12]. Experimentally infected ferrets were euthanized on day 4 of the experiment, and all other animals were euthanized on day 14 of the experiment. Viral titres in nasal wash samples were determined by qPCR [11] and $TCID_{50}$ assay [8].

Figure 1 presents an overall schematic for the serial transmission experiments in the ferret model.

Serial Transmission of virus library in MDCK-SIAT-TMPRSS2 cells

$1x10^5$ MDCK-SIAT-TMPRSS2 cells were seeded in 24-well plates and grown for 24 hours in GIBCO DMEM High Glucose® (Life technologies, US) supplemented with 10% (v/v) foetal bovine serum (JRH Biosciences, US), 2 µM L-glutamine (SAFC Biosciences, US), 200 U/mL penicillin (Sigma-Aldrich, US), 200 µg/mL streptomycin (Sigma-Aldrich, US), and 0.02M HEPES(SAFC Biosciences). After 24 hours, the cells were infected either with the combined virus library in triplicate or the control virus in singlicate at an MOI of 0.1 and incubated in

serum-free media with all supplements and 0.5ug/ml of TPCK-Trypsin (Worthingston, USA). Supernatants were collected at 2 hours and 72 hours post infection and titrated by $TCID_{50}$ on MDCK-SIAT-TMRPSS2 cells to ensure viral growth (Titres greater 10^4 $TCID_{50}$ was observed in all samples after 72 hours). The supernatant at 72 hours post infection was used for RNA extraction and deep sequencing as described below.

Deep sequencing analysis of virus library and ferret nasal washes

The plasmid libraries (i, ii and iii) and virus libraries (i, ii and iii) were deep sequenced, alongside the H275Y-NA plasmid (control for PCR error rate) and the SA16-H275Y virus (control for reverse genetics). A single nasal wash sample was picked from each ferret in the transmission chain for deep sequencing (sample selection is denoted in Figure 4). Factors taken into consideration when selecting nasal wash samples for deep sequencing were (i) selection of time points as late as possible during infection to allow time for within-host selection of variants from the viral mixtures, and (ii) that the RNA quantity and quality was sufficient for deep sequencing and accurate variant calling [13]. Supernatant collected at 72 hours post infection after *in vitro* infection was used for deep sequencing analysis.

Viral RNA from ferret nasal wash samples, and from supernatant after *in vitro* passaging was quantified using qPCR with primers that detect the M gene of influenza A viruses, provided by the US Centers for Disease Control and Prevention, Atlanta, USA.

Viral RNA was extracted from virus library, ferret nasal wash samples, and supernatant after *in vitro* passaging using the QIAamp® Viral RNA mini kit (Qiagen, Germany). Next generation sequencing was carried out twice on the ferret nasal washes: a) on the NA gene only to get a high degree of coverage for analysis and b) for the full genome of the virus to track changes in the internal genes. For the NA gene, cDNA synthesis was carried out using NA gene-specific primers (supplementary text S1), and the SuperScript III First-Strand Synthesis System

(Invitrogen, USA). The NA gene was amplified from the cDNA and plasmids using gene-specific primers and the Platinum™ *Taq* DNA Polymerase High Fidelity kit (Invitrogen, USA) and sent for sequencing. The full genome sequencing was done after amplification of all genes using primers previously described [14]. Sequencing of amplified PCR products were done at the Australian Genome Research Facility, on the HiSeq 2500 platform (2x 150 PE reads, 15 million reads per sample).

Analysis of deep sequencing data and bioinformatics

The NA genes from the plasmid and virus libraries were deep sequenced alongside their respective controls to confirm that all single amino acid substitutions were represented in each library. The mapmuts pipeline (http://jbloom.github.io/mapmuts/) was used to generate codon counts for each site. Codon identities were called only in overlapping regions of the paired-end reads, where both reads concurred. This was done to reduce the sequencing error rate, as the same sequencing error is unlikely to occur in both reads. The dms_tools2 and mapmuts pipelines were then used to confirm the completeness of the libraries. The pipelines were also used to map overlapping fastq reads from ferret nasal washes, and *in vitro* passaging to template NA [1-3, 6, 15].

For ferret nasal wash samples and *in vitro* passaging samples, fastq reads were also mapped to the influenza genome using Bowtie2 v2.2.5 (-very-sensitive-local) (http://bowtiebio.sourceforge.net/index.shtml). SAM tools v1.7 was used to process sequence alignments and generate pileup files. The pileup files were then used to scan for minorities using Varscan [16] with a minimum variant calling threshold set at 1%. The nucleotide diversity and ratio of synonymous to non-synonymous mutations in ferret nasal wash samples was calculating by measuring π and πS/πN using the SNPgenie software [17]. The nucleotide mutation frequencies in donor:recipient pairs from the eight contact transmission pairs and for

aerosol transmission pairs were also used to estimate transmission bottleneck sizes using the beta-binomial sampling method developed by Leonard *et al* [18]. This statistical method takes the stochastic dynamics of viral replication in recipients into account and further considers variant calling thresholds. For our analysis, a minimum variant calling threshold of 1% was utilised to estimate bottleneck size to include a greater number of sites, as was done by Poon *et al.* in a human household transmission study [19]. A more conservative estimate of the bottleneck size was also calculated, using a minimum variant calling threshold of 3% similar to Leonard *et al.* [18].

The dms_tools2 and mapmuts pipelines developed within the laboratory of Dr. Jesse Bloom (Fred Hutchinson Cancer Research Center, Washington, USA) were also used to produce codon-count files, which were then used for amino acid preference analysis [1-3, 6, 15]. Amino acid preferences were used to calculate Shannon entropy, which is a measure of mutational tolerance of the NA protein in experimental settings. Shannon entropy was also calculated for all natural sequences with the H275Y mutation in the GISAID database, after sequence clean-up and alignment using MAFFT [20]. Pymol (Schrödinger, USA) [21] was used to visualize mutational tolerance on the protein structure of the NA from the A(H1N1)pdm09 virus A/California/A/2009 (pdb:4B7R) [22].

References:

1. Thyagarajan, B. and J.D. Bloom, *The inherent mutational tolerance and antigenic evolvability of influenza hemagglutinin.* Elife, 2014. **8**(3): p. 03300.
2. Bloom, J.D., *An experimentally determined evolutionary model dramatically improves phylogenetic fit.* Mol Biol Evol, 2014. **31**(8): p. 1956-78.
3. Doud, M.B. and J.D. Bloom, *Accurate Measurement of the Effects of All Amino-Acid Mutations on Influenza Hemagglutinin.* Viruses, 2016. **8**(6).
4. Hoffmann, E., et al., *Universal primer set for the full-length amplification of all influenza A viruses.* Arch Virol, 2001. **146**(12): p. 2275-89.
5. Hoffmann, E., et al., *A DNA transfection system for generation of influenza A virus from eight plasmids.* Proc Natl Acad Sci U S A, 2000. **97**(11): p. 6108-13.

6. Lee, J.M., et al., *Deep mutational scanning of hemagglutinin helps predict evolutionary fates of human H3N2 influenza variants.* Proc Natl Acad Sci U S A, 2018. **115**(35): p. E8276-E8285.
7. Hirst, G.K., *THE QUANTITATIVE DETERMINATION OF INFLUENZA VIRUS AND ANTIBODIES BY MEANS OF RED CELL AGGLUTINATION.* The Journal of experimental medicine, 1942. **75**(1): p. 49-64.
8. Ramakrishnan, M.A., *Determination of 50% endpoint titer using a simple formula.* World journal of virology, 2016. **5**(2): p. 85-86.
9. Pedersen, J.C., *Hemagglutination-inhibition assay for influenza virus subtype identification and the detection and quantitation of serum antibodies to influenza virus.* Methods Mol Biol, 2014: p. 0758-8_2.
10. Oh, D.Y. and A.C. Hurt, *Using the ferret as an animal model for investigating influenza antiviral effectiveness.* Frontiers in Microbiology, 2016. **7**.
11. Butler, J., et al., *Estimating the fitness advantage conferred by permissive neuraminidase mutations in recent oseltamivir-resistant A(H1N1)pdm09 influenza viruses.* PLoS Pathog, 2014. **10**(4).
12. Oh, D.Y., et al., *Evaluation of oseltamivir prophylaxis regimens for reducing influenza virus infection, transmission and disease severity in a ferret model of household contact.* J Antimicrob Chemother, 2014. **69**(9): p. 2458-69.
13. McCrone, J.T. and A.S. Lauring, *Measurements of Intrahost Viral Diversity Are Extremely Sensitive to Systematic Errors in Variant Calling.* J Virol, 2016. **90**(15): p. 6884-95.
14. Zhou, B., et al., *Single-reaction genomic amplification accelerates sequencing and vaccine production for classical and Swine origin human influenza a viruses.* Journal of virology, 2009. **83**(19): p. 10309-10313.
15. Bloom, J.D., L.I. Gong, and D. Baltimore, *Permissive Secondary Mutations Enable the Evolution of Influenza Oseltamivir Resistance.* Science, 2010. **328**(5983): p. 1272-1275.
16. Koboldt, D.C., et al., *VarScan: variant detection in massively parallel sequencing of individual and pooled samples.* Bioinformatics (Oxford, England), 2009. **25**(17): p. 2283-2285.
17. Nelson, C.W., L.H. Moncla, and A.L. Hughes, *SNPGenie: estimating evolutionary parameters to detect natural selection using pooled next-generation sequencing data.* Bioinformatics (Oxford, England), 2015. **31**(22): p. 3709-11.
18. Sobel Leonard, A., et al., *Transmission Bottleneck Size Estimation from Pathogen Deep-Sequencing Data, with an Application to Human Influenza A Virus.* Journal of Virology, 2017. **91**(14): p. e00171-17.
19. Poon, L.L., et al., *Quantifying influenza virus diversity and transmission in humans.* Nat Genet, 2016. **48**(2): p. 195-200.
20. Katoh, K. and D.M. Standley, *MAFFT multiple sequence alignment software version 7: improvements in performance and usability.* Molecular biology and evolution, 2013. **30**(4): p. 772-780.
21. Delano, W., L, *PyMol*, D. Scientific, Editor. 2002, DeLano Scientific, San Carlos, CA, 700.
22. van der Vries, E., et al., *H1N1 2009 pandemic influenza virus: resistance of the I223R neuraminidase mutant explained by kinetic and structural analysis.* PLoS Pathog, 2012. **8**(9): p. 20.
